# Supplementary material for: Single-cell RNA-seq reveals dynamic transcriptome profiling in human early neural differentiation
Source: Gigascience. 2018 Sep 18;7(11):giy117. doi: 10.1093/gigascience/giy117 (PMC6420650; doi:10.1093/gigascience/giy117)
Supplement: Supplemental Files [file giy117_supplemental_files.zip › Additional file 21_Table S3.pdf]

Contents

EB 2
Numbers of receptor and ligand 2
Interactions 2
GO terms annotation 2
Ros-E 9
Numbers of receptor and ligand 9
Interactions 9
GO terms annotation 9
Ros-L 13
Numbers of receptor and ligand 13
Interactions 13
GO terms annotation 13
NPCs 17
Numbers of receptor and ligand 17
Interactions 17
GO terms annotation 17

EB

Numbers of receptor and ligand

EB	Receptors	Ligands	Total
**Cluster_1**	70	60	130
**Cluster_2**	41	54	95
**Cluster_3**	70	65	135
**Total**	181	179	360

Interactions

Receptors / Ligands	Cluster_1	Cluster_2	Cluster_3	Total ligands
**Cluster_1**		40	69	109
**Cluster_2**	40		47	87
**Cluster_3**	65	43		108
**Total receptor**	105	83	116	304

GO terms annotation

ENSG00000078401	EDN1	neural crest cell development	"The process aimed at the progression of a neural crest cell over time, from initial commitment of the cell to its specific fate, to the fully functional differentiated cell." [GOC:dh, GOC:ef]
ENSG00000162344	FGF19	neural crest cell migration	"The characteristic movement of cells from the dorsal ridge of the neural tube to a variety of locations in a vertebrate embryo." [GOC:ascb_2009, GOC:dph, GOC:tb,

			ISBN:0878932437]
ENSG00000169242	EFNA1	notochord formation	"The formation of the notochord from the chordamesoderm. The notochord is composed of large cells packed within a firm connective tissue sheath and is found in all chordates at the ventral surface of the neural tube. In vertebrates, the notochord contributes to the vertebral column." [GOC:dh, GOC:ef]
ENSG00000136160	EDNRB	neural crest cell migration	"The characteristic movement of cells from the dorsal ridge of the neural tube to a variety of locations in a vertebrate embryo." [GOC:ascb_2009, GOC:dph, GOC:tb, ISBN:0878932437]
ENSG00000064300	NGFR	negative regulation of neuron projection development	"Any process that decreases the rate, frequency or extent of neuron projection development. Neuron projection development is the process whose specific outcome is the progression of a neuron projection over time, from its formation to the mature structure. A neuron projection is any process extending from a neural cell, such as axons or dendrites (collectively called neurites)." [GOC:dph, GOC:tb]
ENSG00000107831	FGF8	neural plate morphogenesis	"The process in which the anatomical structures of the neural plate are generated and organized. The neural plate is a specialized region of columnar epithelial cells in the dorsal ectoderm that will give rise to nervous system tissue." [GOC:dph,

			ISBN:0878932437]
ENSG00000114251	WNT5A	positive regulation of neuron projection development	"Any process that increases the rate, frequency or extent of neuron projection development. Neuron projection development is the process whose specific outcome is the progression of a neuron projection over time, from its formation to the mature structure. A neuron projection is any process extending from a neural cell, such as axons or dendrites (collectively called neurites)." [GOC:dph, GOC:tb]
ENSG00000125266	EFNB2	negative regulation of neuron projection development	"Any process that decreases the rate, frequency or extent of neuron projection development. Neuron projection development is the process whose specific outcome is the progression of a neuron projection over time, from its formation to the mature structure. A neuron projection is any process extending from a neural cell, such as axons or dendrites (collectively called neurites)." [GOC:dph, GOC:tb]
ENSG00000180340	FZD2	planar cell polarity pathway involved in neural tube closure	"The series of molecular signals initiated by binding of a Wnt protein to a receptor on the surface of the target cell where activated receptors signal via downstream effectors that modulates the establishment of planar polarity contributing to neural tube closure." [GOC:ascb_2009, GOC:dph, GOC:tb]
ENSG00000101384	JAG1	cardiac neural	"The process aimed at the

		crest cell development involved in outflow tract morphogenesis	progression of a cardiac neural crest cell over time, from initial commitment of the cell to its specific fate, to the fully functional differentiated cell that contributes to the shaping of the outflow tract." [GOC:dph, GOC:mtg_heart]
ENSG00000090932	DLL3	paraxial mesoderm development	"The process whose specific outcome is the progression of the paraxial mesoderm over time, from its formation to the mature structure. The paraxial mesoderm is the mesoderm located bilaterally adjacent to the notochord and neural tube." [GOC:dgh]
ENSG00000090776	EFNB1	neural crest cell migration	"The characteristic movement of cells from the dorsal ridge of the neural tube to a variety of locations in a vertebrate embryo." [GOC:ascb_2009, GOC:dph, GOC:tb, ISBN:0878932437]
ENSG00000101144	BMP7	neural fold elevation formation	"The process in which the lateral borders of the neural plate begin to migrate upwards to form the neural folds, caused by the proliferation of the underlying mesoderm." [GO_REF:0000021, GOC:cls, GOC:dgh, GOC:dph, GOC:jid, GOC:mtg_15jun06, PMID:15806586]
ENSG00000164930	FZD6	neural tube closure	"The last step in the formation of the neural tube, where the paired neural folds are brought together and fuse at the dorsal midline." [GOC:dph, ISBN:0878932437]
ENSG00000115170	ACVR1	neural crest cell migration	"The characteristic movement of cells from the dorsal ridge of the neural tube to a variety of locations in a vertebrate

			embryo." [GOC:ascb_2009, GOC:dph, GOC:tb, ISBN:0878932437]
ENSG00000107779	BMPR1A	neural crest cell development	"The process aimed at the progression of a neural crest cell over time, from initial commitment of the cell to its specific fate, to the fully functional differentiated cell." [GOC:dh, GOC:ef]
ENSG00000185652	NTF3	neuron projection morphogenesis	"The process in which the anatomical structures of a neuron projection are generated and organized. A neuron projection is any process extending from a neural cell, such as axons or dendrites." [GOC:mah]
ENSG00000077782	FGFR1	chordate embryonic development	"The process whose specific outcome is the progression of the embryo over time, from zygote formation through a stage including a notochord and neural tube until birth or egg hatching." [GOC:mtg_sensu]
ENSG00000116106	EPHA4	negative regulation of neuron projection development	"Any process that decreases the rate, frequency or extent of neuron projection development. Neuron projection development is the process whose specific outcome is the progression of a neuron projection over time, from its formation to the mature structure. A neuron projection is any process extending from a neural cell, such as axons or dendrites (collectively called neurites)." [GOC:dph, GOC:tb]
ENSG00000170989	S1PR1	brain development	"The process whose specific outcome is the progression of the brain over time, from its

			formation to the mature structure. Brain development begins with patterning events in the neural tube and ends with the mature structure that is the center of thought and emotion. The brain is responsible for the coordination and control of bodily activities and the interpretation of information from the senses (sight, hearing, smell, etc.)." [GOC:dph, GOC:jid, GOC:tb, UBERON:0000955]
ENSG00000135333	EPHA7	brain development	"The process whose specific outcome is the progression of the brain over time, from its formation to the mature structure. Brain development begins with patterning events in the neural tube and ends with the mature structure that is the center of thought and emotion. The brain is responsible for the coordination and control of bodily activities and the interpretation of information from the senses (sight, hearing, smell, etc.)." [GOC:dph, GOC:jid, GOC:tb, UBERON:0000955]
ENSG00000104290	FZD3	neural tube closure	"The last step in the formation of the neural tube, where the paired neural folds are brought together and fuse at the dorsal midline." [GOC:dph, ISBN:0878932437]
ENSG00000142627	EPHA2	notochord formation	"The formation of the notochord from the chordamesoderm. The notochord is composed of large cells packed within a

			firm connective tissue sheath and is found in all chordates at the ventral surface of the neural tube. In vertebrates, the notochord contributes to the vertebral column." [GOC:dh, GOC:ef]
ENSG00000105894	PTN	brain development	"The process whose specific outcome is the progression of the brain over time, from its formation to the mature structure. Brain development begins with patterning events in the neural tube and ends with the mature structure that is the center of thought and emotion. The brain is responsible for the coordination and control of bodily activities and the interpretation of information from the senses (sight, hearing, smell, etc.)." [GOC:dph, GOC:jid, GOC:tb, UBERON:0000955]

Ros-E

Numbers of receptor and ligand

Ros-E(182, 55)	Receptor	Ligand	total
**Cluster_1**	35	59	94
**Cluster_2**	34	54	88
**Total**	69	113	182

Interactions

Receptor / Ligand	Cluster_1	Cluster_2	Total ligand
**Cluster_1**		29	29
**Cluster_2**	26		26
**Total receptor**	26	29	55

GO terms annotation

ENSG00000078401	EDN1	neural crest cell development	"The process aimed at the progression of a neural crest cell over time, from initial commitment of the cell to its specific fate, to the fully functional differentiated cell." [GOC:dh, GOC:ef]
ENSG00000162344	FGF19	neural crest cell migration	"The characteristic movement of cells from the dorsal ridge of the neural tube to a variety of locations in a vertebrate embryo." [GOC:ascb_2009, GOC:dph, GOC:tb, ISBN:0878932437]
ENSG00000169242	EFNA1	notochord formation	"The formation of the notochord from the chordamesoderm. The notochord is composed of large

			cells packed within a firm connective tissue sheath and is found in all chordates at the ventral surface of the neural tube. In vertebrates, the notochord contributes to the vertebral column." [GOC:dh, GOC:ef]
ENSG00000136160	EDNRB	neural crest cell migration	"The characteristic movement of cells from the dorsal ridge of the neural tube to a variety of locations in a vertebrate embryo." [GOC:ascb_2009, GOC:dph, GOC:tb, ISBN:0878932437]
ENSG00000114251	WNT5A	positive regulation of neuron projection development	"Any process that increases the rate, frequency or extent of neuron projection development. Neuron projection development is the process whose specific outcome is the progression of a neuron projection over time, from its formation to the mature structure. A neuron projection is any process extending from a neural cell, such as axons or dendrites (collectively called neurites)." [GOC:dph, GOC:tb]
ENSG00000125266	EFNB2	negative regulation of neuron projection development	"Any process that decreases the rate, frequency or extent of neuron projection development. Neuron projection development is the process whose specific outcome is the progression of a neuron projection over time, from its formation to the mature structure. A neuron projection is any process extending from a neural cell, such as axons or dendrites (collectively called neurites)." [GOC:dph, GOC:tb]
ENSG00000180340	FZD2	planar cell polarity pathway	"The series of molecular signals initiated by binding of a Wnt protein to a receptor on the

		involved in neural tube closure	surface of the target cell where activated receptors signal via downstream effectors that modulates the establishment of planar polarity contributing to neural tube closure." [GOC:ascb_2009, GOC:dph, GOC:tb]
ENSG00000090932	DLL3	paraxial mesoderm development	"The process whose specific outcome is the progression of the paraxial mesoderm over time, from its formation to the mature structure. The paraxial mesoderm is the mesoderm located bilaterally adjacent to the notochord and neural tube." [GOC:dgh]
ENSG00000125378	BMP4	neural tube closure	"The last step in the formation of the neural tube, where the paired neural folds are brought together and fuse at the dorsal midline." [GOC:dph, ISBN:0878932437]
ENSG00000164930	FZD6	neural tube closure	"The last step in the formation of the neural tube, where the paired neural folds are brought together and fuse at the dorsal midline." [GOC:dph, ISBN:0878932437]
ENSG00000077782	FGFR1	positive regulation of neuron differentiation	"Any process that activates or increases the frequency, rate or extent of neuron differentiation." [GOC:go_curators]
ENSG00000116106	EPHA4	negative regulation of neuron projection development	"Any process that decreases the rate, frequency or extent of neuron projection development. Neuron projection development is the process whose specific outcome is the progression of a neuron projection over time, from its formation to the mature structure. A neuron projection is any process extending from a neural cell, such as axons or dendrites (collectively called neurites)." [GOC:dph, GOC:tb]

ENSG00000170989	S1PR1	brain development	"The process whose specific outcome is the progression of the brain over time, from its formation to the mature structure. Brain development begins with patterning events in the neural tube and ends with the mature structure that is the center of thought and emotion. The brain is responsible for the coordination and control of bodily activities and the interpretation of information from the senses (sight, hearing, smell, etc.)." [GOC:dph, GOC:jid, GOC:tb, UBERON:0000955]
ENSG00000104290	FZD3	neural tube closure	"The last step in the formation of the neural tube, where the paired neural folds are brought together and fuse at the dorsal midline." [GOC:dph, ISBN:0878932437]
ENSG00000142627	EPHA2	notochord formation	"The formation of the notochord from the chordamesoderm. The notochord is composed of large cells packed within a firm connective tissue sheath and is found in all chordates at the ventral surface of the neural tube. In vertebrates, the notochord contributes to the vertebral column." [GOC:dh, GOC:ef]

Ros-L

Numbers of receptor and ligand

Ros-L	Receptor	Ligand	Total
**Cluster_1**	31	56	87
**Cluster_2**	32	47	79
**Cluster_3**	34	61	95
**Total**	97	164	261

Interactions

Receptor / Ligand	Cluster_1	Cluster_2	Cluster_3	Total ligand
**Cluster_1**		27	25	52
**Cluster_2**	13		17	30
**Cluster_3**	19	23		42
**Total receptor**	32	50	42	124

GO terms annotation

ENSG00000078401	EDN1	neural crest cell development	"The process aimed at the progression of a neural crest cell over time, from initial commitment of the cell to its specific fate, to the fully functional differentiated cell." [GOC:dh, GOC:ef]
ENSG00000099250	NRP1	neural crest cell migration involved in autonomic nervous	"Any neural crest cell migration that is involved in autonomic nervous system development." [GOC:BHF, GOC:TermGenie]

		system development	
ENSG00000114251	WNT5A	positive regulation of neuron projection development	"Any process that increases the rate, frequency or extent of neuron projection development. Neuron projection development is the process whose specific outcome is the progression of a neuron projection over time, from its formation to the mature structure. A neuron projection is any process extending from a neural cell, such as axons or dendrites (collectively called neurites)." [GOC:dph, GOC:tb]
ENSG00000180340	FZD2	planar cell polarity pathway involved in neural tube closure	"The series of molecular signals initiated by binding of a Wnt protein to a receptor on the surface of the target cell where activated receptors signal via downstream effectors that modulates the establishment of planar polarity contributing to neural tube closure." [GOC:ascb_2009, GOC:dph, GOC:tb]
ENSG00000151617	EDNRA	enteric nervous system development	"The process whose specific outcome is the progression of the enteric nervous system over time, from its formation to the mature structure. The enteric nervous system is composed of two ganglionated neural plexuses in the gut wall which form one of the three major divisions of the autonomic nervous system. The enteric nervous system innervates the gastrointestinal tract, the pancreas, and the gall bladder. It contains sensory neurons, interneurons, and motor neurons. Thus the circuitry can autonomously sense the tension and the chemical environment in

			the gut and regulate blood vessel tone, motility, secretions, and fluid transport. The system is itself governed by the central nervous system and receives both parasympathetic and sympathetic innervation." [FMA:66070, GOC:jid, GOC:sr]
ENSG00000125378	BMP4	neural tube closure	"The last step in the formation of the neural tube, where the paired neural folds are brought together and fuse at the dorsal midline." [GOC:dph, ISBN:0878932437]
ENSG00000148926	ADM	neural tube closure	"The last step in the formation of the neural tube, where the paired neural folds are brought together and fuse at the dorsal midline." [GOC:dph, ISBN:0878932437]
ENSG00000187266	EPOR	brain development	"The process whose specific outcome is the progression of the brain over time, from its formation to the mature structure. Brain development begins with patterning events in the neural tube and ends with the mature structure that is the center of thought and emotion. The brain is responsible for the coordination and control of bodily activities and the interpretation of information from the senses (sight, hearing, smell, etc.)." [GOC:dph, GOC:jid, GOC:tb, UBERON:0000955]
ENSG00000157240	FZD1	planar cell polarity pathway involved in neural tube closure	"The series of molecular signals initiated by binding of a Wnt protein to a receptor on the surface of the target cell where activated receptors signal via downstream effectors that modulates the establishment of planar polarity contributing to neural tube closure." [GOC:ascb_2009, GOC:dph,

			GOC:tb]
ENSG00000077782	FGFR1	chordate embryonic development	"The process whose specific outcome is the progression of the embryo over time, from zygote formation through a stage including a notochord and neural tube until birth or egg hatching." [GOC:mtg_sensu]
ENSG00000170989	S1PR1	brain development	"The process whose specific outcome is the progression of the brain over time, from its formation to the mature structure. Brain development begins with patterning events in the neural tube and ends with the mature structure that is the center of thought and emotion. The brain is responsible for the coordination and control of bodily activities and the interpretation of information from the senses (sight, hearing, smell, etc.)." [GOC:dph, GOC:jid, GOC:tb, UBERON:0000955]

NPCs

Numbers of receptor and ligand

NPCs	Receptor	Ligand	Total
**Cluster_1**	40	55	95
**Cluster_2**	44	61	105
**Cluster_3**	44	63	107
**Total**	128	179	307

Interactions

Receptor / Ligand	Cluster_1	Cluster_2	Cluster_3	Total ligand
**Cluster_1**		26	31	57
**Cluster_2**	24		34	58
**Cluster_3**	23	24		47
**Total receptor**	47	50	65	162

GO terms annotation

ENSG00000078401	EDN1	neural crest cell development	"The process aimed at the progression of a neural crest cell over time, from initial commitment of the cell to its specific fate, to the fully functional differentiated cell." [GOC:dh, GOC:ef]
		rhythmic excitation	"Any process involved in the generation of rhythmic, synchronous excitatory

			synaptic inputs in a neural circuit." [GOC:go_curators, ISBN:0195088433]
ENSG00000169242	EFNA1	notochord formation	"The formation of the notochord from the chordamesoderm. The notochord is composed of large cells packed within a firm connective tissue sheath and is found in all chordates at the ventral surface of the neural tube. In vertebrates, the notochord contributes to the vertebral column." [GOC:dh, GOC:ef]
ENSG00000092969	TGFB2	neural tube closure	"The last step in the formation of the neural tube, where the paired neural folds are brought together and fuse at the dorsal midline." [GOC:dph, ISBN:0878932437]
ENSG00000114251	WNT5A	positive regulation of neuron projection development	"Any process that increases the rate, frequency or extent of neuron projection development. Neuron projection development is the process whose specific outcome is the progression of a neuron projection over time, from its formation to the mature structure. A neuron projection is any process extending from a neural cell, such as axons or dendrites (collectively called neurites)." [GOC:dph, GOC:tb]
		neural tube closure	"The last step in the formation of the neural tube, where the paired

			neural folds are brought together and fuse at the dorsal midline." [GOC:dph, ISBN:0878932437]
		neural tube development	"The process whose specific outcome is the progression of the neural tube over time, from its formation to the mature structure. The mature structure of the neural tube exists when the tube has been segmented into the forebrain, midbrain, hindbrain and spinal cord regions. In addition neural crest has budded away from the epithelium." [GO_REF:0000021, GOC:cls, GOC:dgh, GOC:dph, GOC:jid, GOC:mtg_15jun06]
		neuron projection morphogenesis	"The process in which the anatomical structures of a neuron projection are generated and organized. A neuron projection is any process extending from a neural cell, such as axons or dendrites." [GOC:mah]
		planar cell polarity pathway involved in neural tube closure	"The series of molecular signals initiated by binding of a Wnt protein to a receptor on the surface of the target cell where activated receptors signal via downstream effectors that modulates the establishment of planar polarity contributing to neural tube closure." [GOC:ascb_2009, GOC:dph, GOC:tb]

		melanocyte proliferation	"The multiplication or reproduction of melanocytes, resulting in the expansion of a cell population. A melanocyte is a pigment cell derived from the neural crest. It contains melanin-filled pigment granules, which give a brown to black appearance." [CL:0000148, GOC:uh, PMID:22637532]
ENSG00000101384	JAG1	cardiac neural crest cell development involved in outflow tract morphogenesis	"The process aimed at the progression of a cardiac neural crest cell over time, from initial commitment of the cell to its specific fate, to the fully functional differentiated cell that contributes to the shaping of the outflow tract." [GOC:dph, GOC:mtg_heart]
ENSG00000090932	DLL3	paraxial mesoderm development	"The process whose specific outcome is the progression of the paraxial mesoderm over time, from its formation to the mature structure. The paraxial mesoderm is the mesoderm located bilaterally adjacent to the notochord and neural tube." [GOC:dgh]
ENSG00000125378	BMP4	neural tube closure	"The last step in the formation of the neural tube, where the paired neural folds are brought together and fuse at the dorsal midline." [GOC:dph, ISBN:0878932437]
		dorsal/ventral neural tube patterning	"The process in which the neural tube is regionalized in the dorsoventral axis."

			[GO_REF:0000021, GOC:cls, GOC:dgh, GOC:dph, GOC:jid, GOC:mtg_15jun06, PMID:11262869]
		positive regulation of cardiac neural crest cell migration	"Any process that activates or increases the frequency, rate or extent of cardiac neural crest cell migration involved in outflow tract morphogenesis." [GO_REF:0000058, GOC:BHF, GOC:rl, GOC:TermGenie, PMID:17628518]
ENSG00000136244	IL6	positive regulation of neuron projection development	"Any process that increases the rate, frequency or extent of neuron projection development. Neuron projection development is the process whose specific outcome is the progression of a neuron projection over time, from its formation to the mature structure. A neuron projection is any process extending from a neural cell, such as axons or dendrites (collectively called neurites)." [GOC:dph, GOC:tb]
		neuron projection development	"The process whose specific outcome is the progression of a neuron projection over time, from its formation to the mature structure. A neuron projection is any process extending from a neural cell, such as axons or dendrites (collectively called neurites)." [GOC:mah]
ENSG00000107562	CXCL12	brain	"The process whose

		development	specific outcome is the progression of the brain over time, from its formation to the mature structure. Brain development begins with patterning events in the neural tube and ends with the mature structure that is the center of thought and emotion. The brain is responsible for the coordination and control of bodily activities and the interpretation of information from the senses (sight, hearing, smell, etc.)." [GOC:dph, GOC:jid, GOC:tb, UBERON:0000955]
ENSG00000099250	NRP1	ventral trunk neural crest cell migration	"The movement of trunk neural crest cells from the neural tube, travelling ventrally through the anterior half of each sclerotome. Trunk neural crest cells that remain in the sclerotome form the dorsal root ganglia containing the sensory neurons. Trunk neural crest cells that continue more ventrally form the sympathetic ganglia, the adrenal medulla, and the nerve clusters surrounding the aorta." [GOC:bf, GOC:mat, GOC:PARL, PMID:16319111, PMID:19386662]
		neural crest cell migration involved in autonomic	"Any neural crest cell migration that is involved in autonomic nervous system development."

		nervous system development	[GOC:BHF, GOC:TermGenie]
ENSG00000136160	EDNRB	neural crest cell migration	"The characteristic movement of cells from the dorsal ridge of the neural tube to a variety of locations in a vertebrate embryo." [GOC:ascb_2009, GOC:dph, GOC:tb, ISBN:0878932437]
		enteric nervous system development	"The process whose specific outcome is the progression of the enteric nervous system over time, from its formation to the mature structure. The enteric nervous system is composed of two ganglionated neural plexuses in the gut wall which form one of the three major divisions of the autonomic nervous system. The enteric nervous system innervates the gastrointestinal tract, the pancreas, and the gall bladder. It contains sensory neurons, interneurons, and motor neurons. Thus the circuitry can autonomously sense the tension and the chemical environment in the gut and regulate blood vessel tone, motility, secretions, and fluid transport. The system is itself governed by the central nervous system and receives both parasympathetic and sympathetic innervation." [FMA:66070, GOC:jid,

			GOC:sr]
ENSG00000180340	FZD2	planar cell polarity pathway involved in neural tube closure	"The series of molecular signals initiated by binding of a Wnt protein to a receptor on the surface of the target cell where activated receptors signal via downstream effectors that modulates the establishment of planar polarity contributing to neural tube closure." [GOC:ascb_2009, GOC:dph, GOC:tb]
ENSG00000151617	EDNRA	enteric nervous system development	"The process whose specific outcome is the progression of the enteric nervous system over time, from its formation to the mature structure. The enteric nervous system is composed of two ganglionated neural plexuses in the gut wall which form one of the three major divisions of the autonomic nervous system. The enteric nervous system innervates the gastrointestinal tract, the pancreas, and the gall bladder. It contains sensory neurons, interneurons, and motor neurons. Thus the circuitry can autonomously sense the tension and the chemical environment in the gut and regulate blood vessel tone, motility, secretions, and fluid transport. The system is itself governed by the central nervous system and receives both

			parasympathetic and sympathetic innervation." [FMA:66070, GOC:jid, GOC:sr]
ENSG00000204217	BMPR2	brain development	"The process whose specific outcome is the progression of the brain over time, from its formation to the mature structure. Brain development begins with patterning events in the neural tube and ends with the mature structure that is the center of thought and emotion. The brain is responsible for the coordination and control of bodily activities and the interpretation of information from the senses (sight, hearing, smell, etc.)." [GOC:dph, GOC:jid, GOC:tb, UBERON:0000955]
ENSG00000164930	FZD6	neural tube closure	"The last step in the formation of the neural tube, where the paired neural folds are brought together and fuse at the dorsal midline." [GOC:dph, ISBN:0878932437]
ENSG00000157240	FZD1	planar cell polarity pathway involved in neural tube closure	"The series of molecular signals initiated by binding of a Wnt protein to a receptor on the surface of the target cell where activated receptors signal via downstream effectors that modulates the establishment of planar polarity contributing to neural tube closure."

			[GOC:ascb_2009, GOC:dph, GOC:tb]
ENSG00000077782	FGFR1	chordate embryonic development	"The process whose specific outcome is the progression of the embryo over time, from zygote formation through a stage including a notochord and neural tube until birth or egg hatching." [GOC:mtg_sensu]
ENSG00000170989	S1PR1	brain development	"The process whose specific outcome is the progression of the brain over time, from its formation to the mature structure. Brain development begins with patterning events in the neural tube and ends with the mature structure that is the center of thought and emotion. The brain is responsible for the coordination and control of bodily activities and the interpretation of information from the senses (sight, hearing, smell, etc.)." [GOC:dph, GOC:jid, GOC:tb, UBERON:0000955]
