# Supplementary material for: Single-cell RNA-seq reveals dynamic transcriptome profiling in human early neural differentiation
Source: Gigascience. 2018 Sep 18;7(11):giy117. doi: 10.1093/gigascience/giy117 (PMC6420650; doi:10.1093/gigascience/giy117)
Supplement: Supplemental Files [file giy117_supplemental_files.zip › Additional files_Aug15_2018.pdf]

Additional file 1: Figure S1

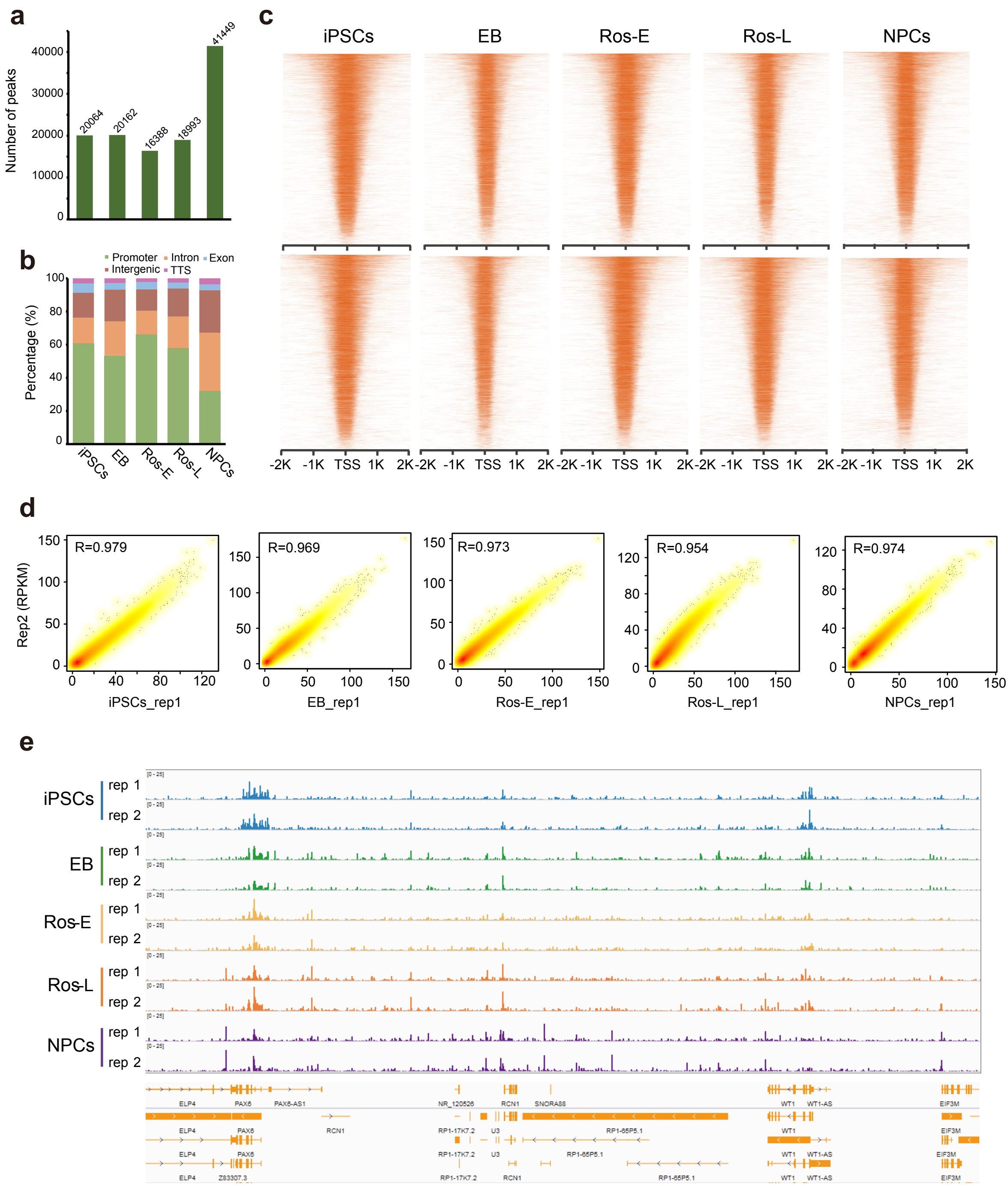

Additional file 1: Figure S1. Quality control of ATAC-seq. a Bar graphs indicate the number of chromatin open regions detected at each cell stage of neural differentiation. **b** Genomic components (distribution) of the peaks in each cell stage during neural differentiation. **c** Heatmaps reporting the chromatin accessibility density within ±2 kb of TSSs. **d** Biological replicates of bulk ATAC-seq show high reproducibility. **e** IGV screenshot showing highly correlated ATAC signals in selected region between replicates.

Additional file 2: Figure S2

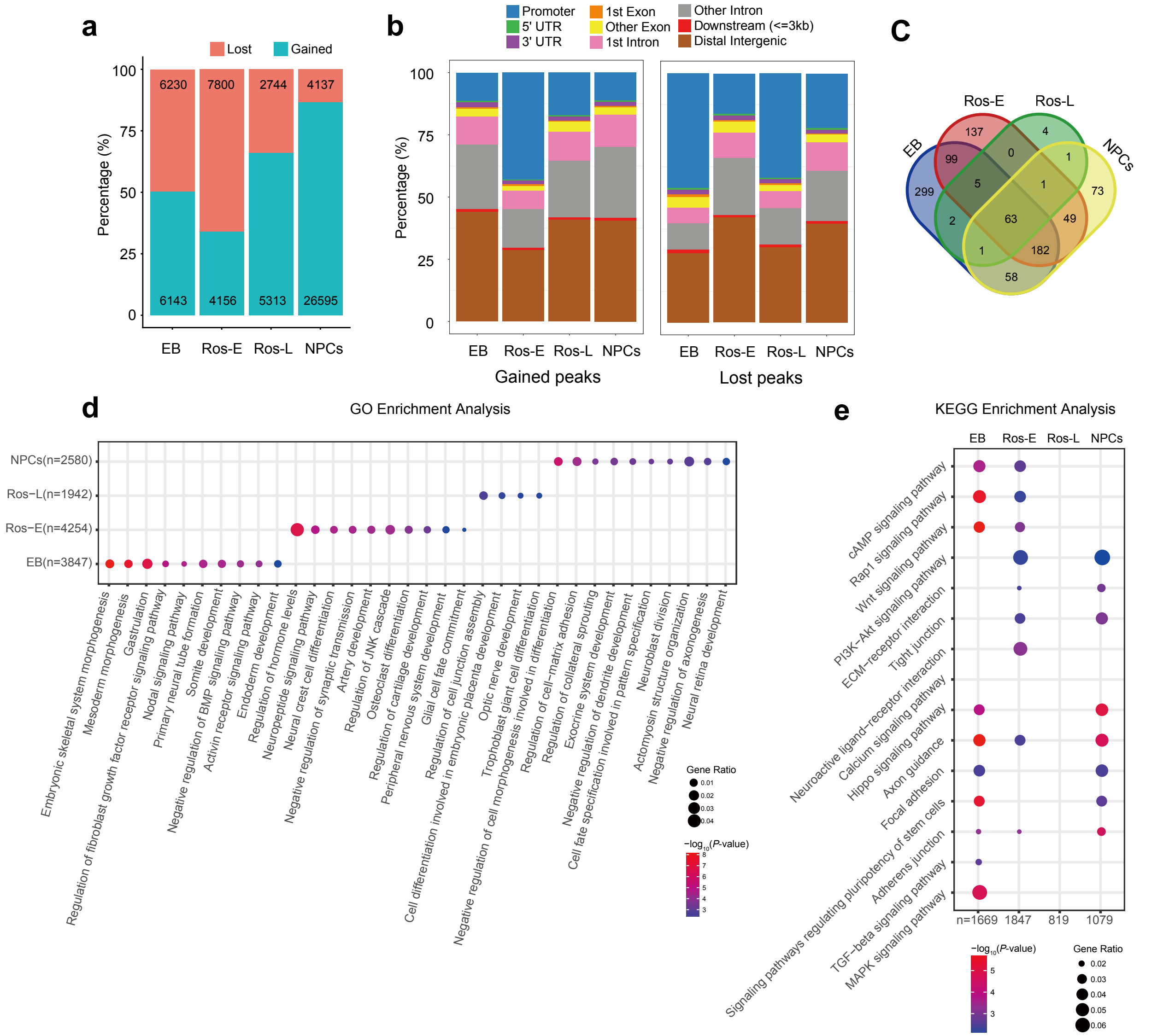

Additional file 2: Figure S2. Dynamics of gained and lost peaks during neural differentiation. a Bar graph shows the number of gained and lost peaks at each cell stage. **b** Bar graph shows genomic composition of gained and lost peaks at each cell stage respectively. **c** Venn plot of GO enrichment analysis on the genes associated with lost peaks at each stage (adjusted *P*-value ≤ 0.01). **d** Selected GO terms identified by genes associated with lost peaks specific to the respective indicated cell stage (adjusted *P*-value ≤ 0.01). **e** Selected differential pathways identified by genes associated with lost peaks at indicated cell stages (adjusted *P*-value ≤ 0.01).

Additional file 3: Figure S3

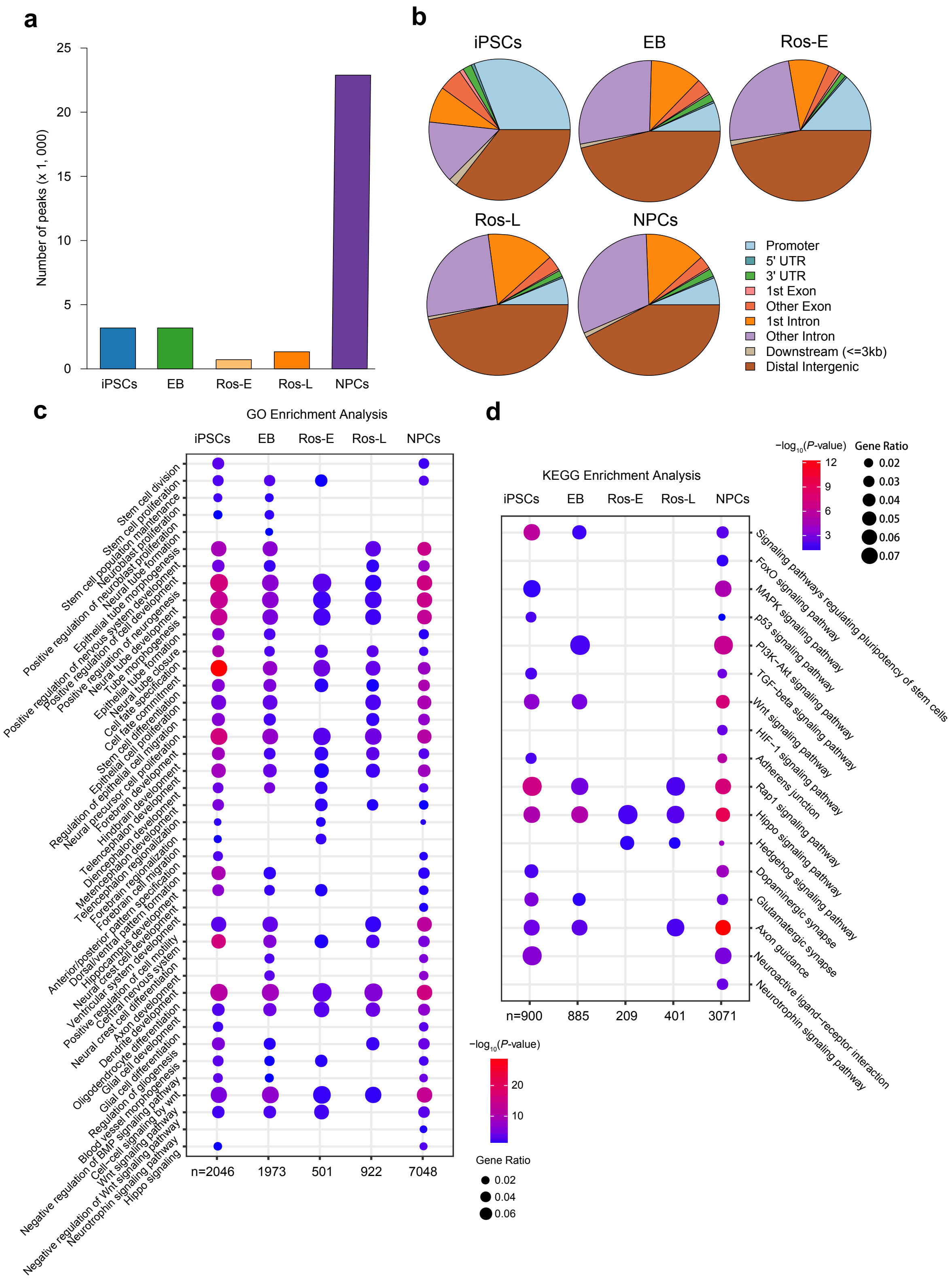

Additional file 3: Figure S3. Stage-specific features of *cis*-regulatory elements during neural differentiation. a Bar plot showing the number of stage specific ATAC peaks at iPSCs, EB, Ros-E, Ros-L and NPCs stage (adjusted *P*-value ≤ 0.01). **b** Pie chart shows genomic composition of stage specific peaks respectively. **c, d** GO term and KEGG enrichment analysis of stage specific peaks, respectively (adjusted *P*-value ≤ 0.05).

Additional file 4: Figure S4

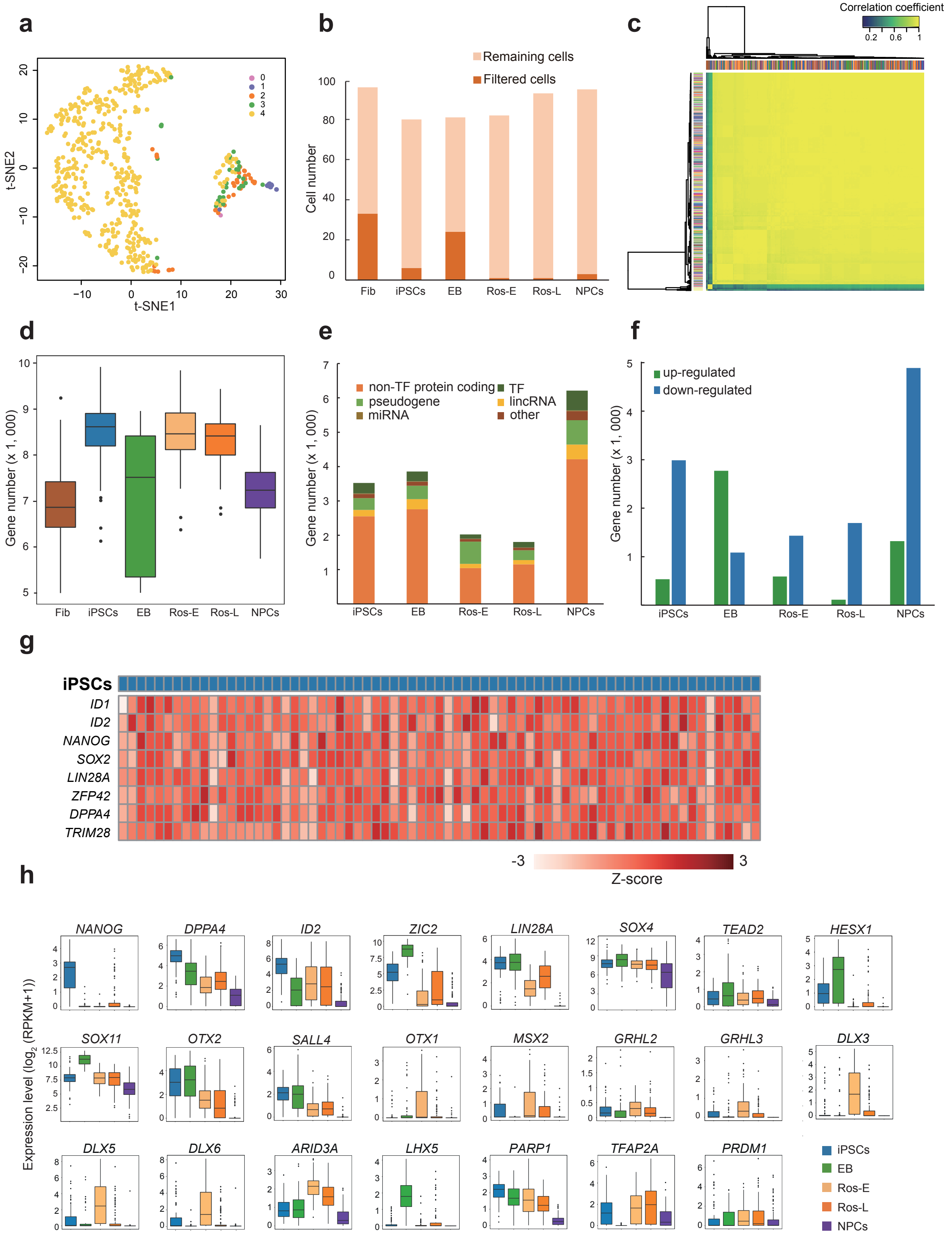

Additional file 4: Figure S4. Quality control of scRNA-seq. a Graph indicates data quality of totally 527 single cells. Color scheme indicates the filter conditions, each dot represents one cell, and yellow dots showing the cells that successfully passed all criteria were used for downstream analysis. **b** Bar plots show the percentage of filtered cells and remaining cells. **c** ERCC correlation analysis of all single cells showing very little batch effects. **d** Box plots report the number of expressed genes for each cell stage after quality control filtering. Each dot represents an outlier gene and each box represents the median and first and third quartiles. **e** Genomic distribution of genes at each cell stage. **f** Summary of up-regulated and down-regulated genes at each cell stage compared to other stages. **g** Expression pattern of pluripotency-associated genes in iPSCs. Color scheme is based on z-score distribution from -3 (light red) to 3 (red). **h** Expression pattern of representative differentially expressed TFs during neural differentiation (adjusted *P*-value ≤ 0.01).

Additional file 5: Figure S5

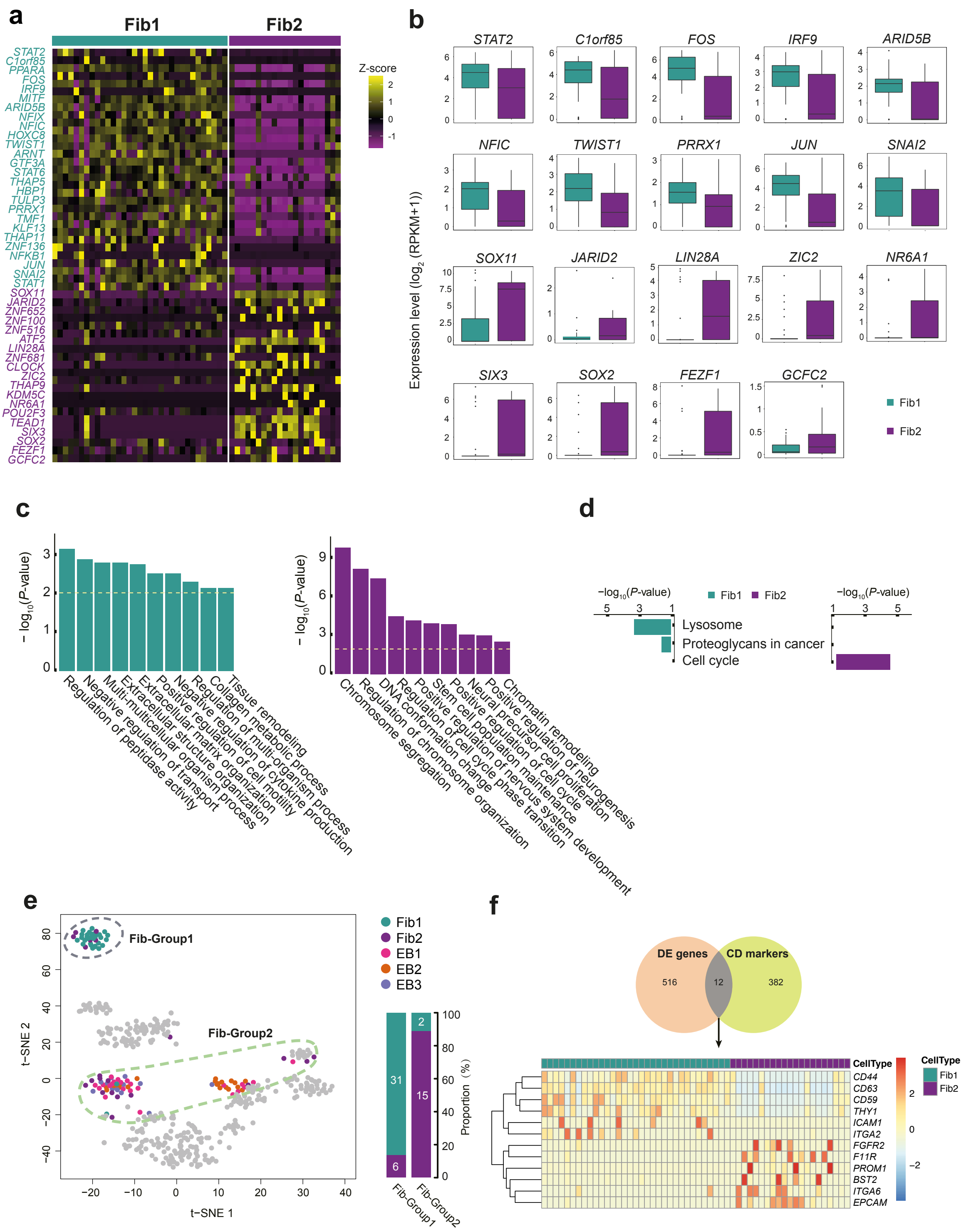

Additional file 5: Figure S5. Subgroups identification and key transcriptomic features within Fib stage. a Heatmap reports scaled expression [log_2(RPKM+1)] of discriminative TF sets for each cluster in Fib stage with *P*-value cutoff ≤ 0.01. Color scheme is based on z-score distribution from -1 (purple) to 2 (yellow). Gene symbols highlight with color specific to the respective Fib subset. **b** Box plots of selected TFs defined in Figure S5a. **c** Selected GO terms identified by up-regulated genes specific to the respective Fib subpopulation with the color as indicated (Green: GO terms specific to Fib1; purple: GO terms specific to Fib2). **d** KEGG enrichment analysis of all terms in Fib subpopulation, respectively. **e** Fib-Group1 and Fib-Group2 based on their location on the t-SNE are marked by dashed ellipse. The columns represent the components of Fib-Group1 and Fib-Group2, respectively. **f** Comparison of differentially expressed (DE) genes between Fib subpopulation with CD markers dataset (HUGO Gene Nomenclature Committee, HGNC) and the heatmap of differentially expressed CD markers between the two Fib subpopulation.

Additional file 6: Figure S6

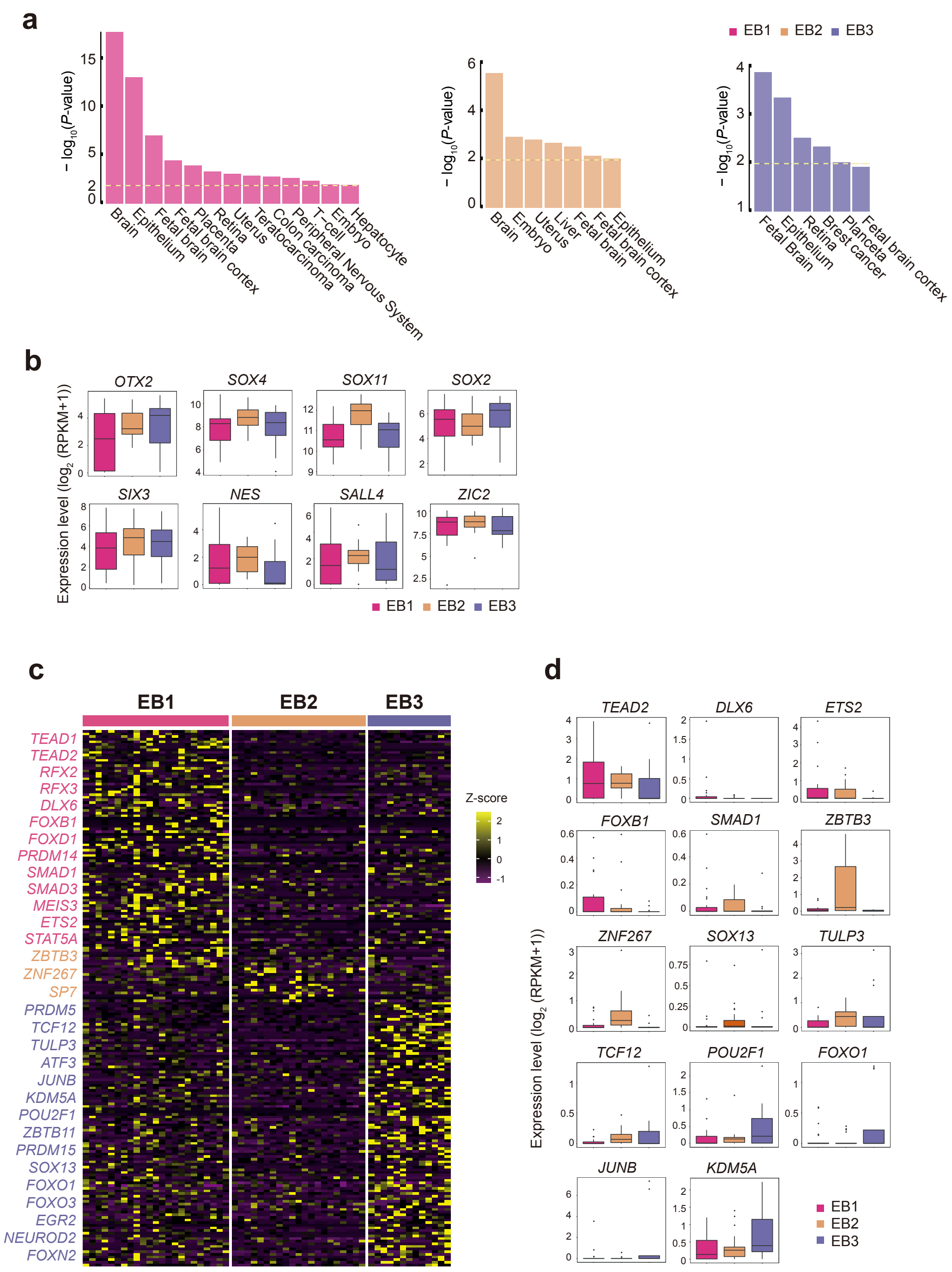

Additional file 6: Figure S6. Subgroups identification and key transcriptomic features within EB stage. a David for tissue enrichment analysis of up-regulated genes defined by three EB subgroups compared to iPSCs stage respectively. **b** Box plots of commonly expressed genes across EB subsets. **c** Heatmap reports scaled expression [$\log_2$(RPKM+1)] of discriminative TF sets for each cluster in EB stage with *P*-value cutoff ≤ 0.01. Color scheme is based on z-score distribution from -1(purple) to 2 (yellow). Gene symbols highlight with color specific to the respective EB subset. **d** Box plot of selected TFs defined in Figure S6c.

Additional file 7: Figure S7

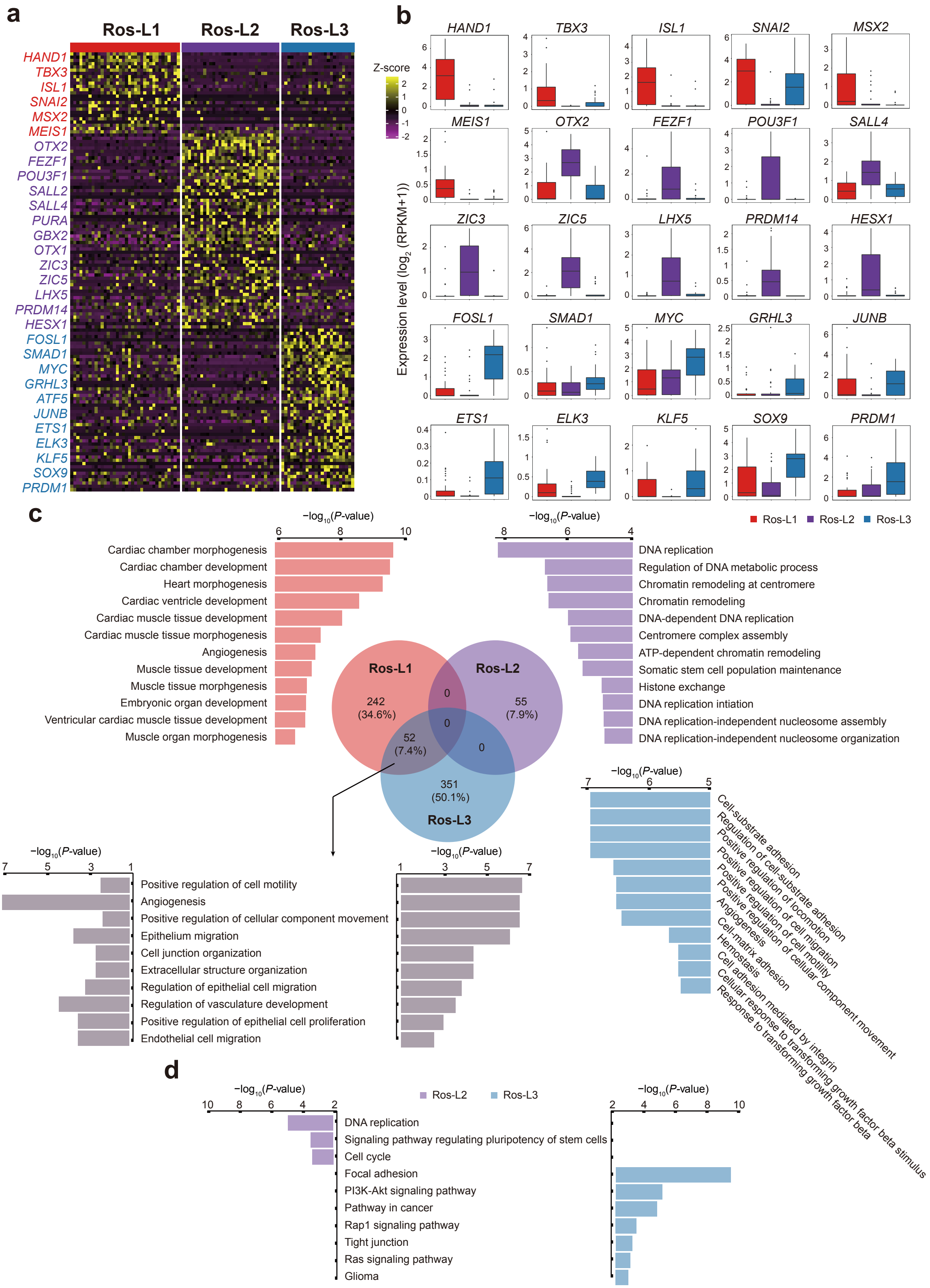

Additional file 7: Figure S7. Subgroups identification and key transcriptomic features within Ros-L stage. a Heatmap reports scaled expression [$\log_2$(RPKM+1)] of discriminative TF sets for each cluster in Ros-L stage with *P*-value cutoff ≤ 0.01. Color scheme is based on z-score distribution from -2 (purple) to 2 (yellow). Gene symbols highlight with color specific to the respective Ros-L subset. **b** Box plots of selected TFs defined in Figure S7a. **c** Top 12 of GO terms identified by up-regulated genes specific to the respective Ros-L subpopulation with the color as indicated (red: GO terms specific to Ros-L1; purple: GO terms specific to Ros-L2; blue: GO terms specific to Ros-L3; gray: selected GO terms shared by Ros-L1 and Ros-L3). **d** KEGG enrichment analysis of Ros-L2 (all terms) and Ros-L3 (selected terms), respectively.

Additional file 8: Figure S8

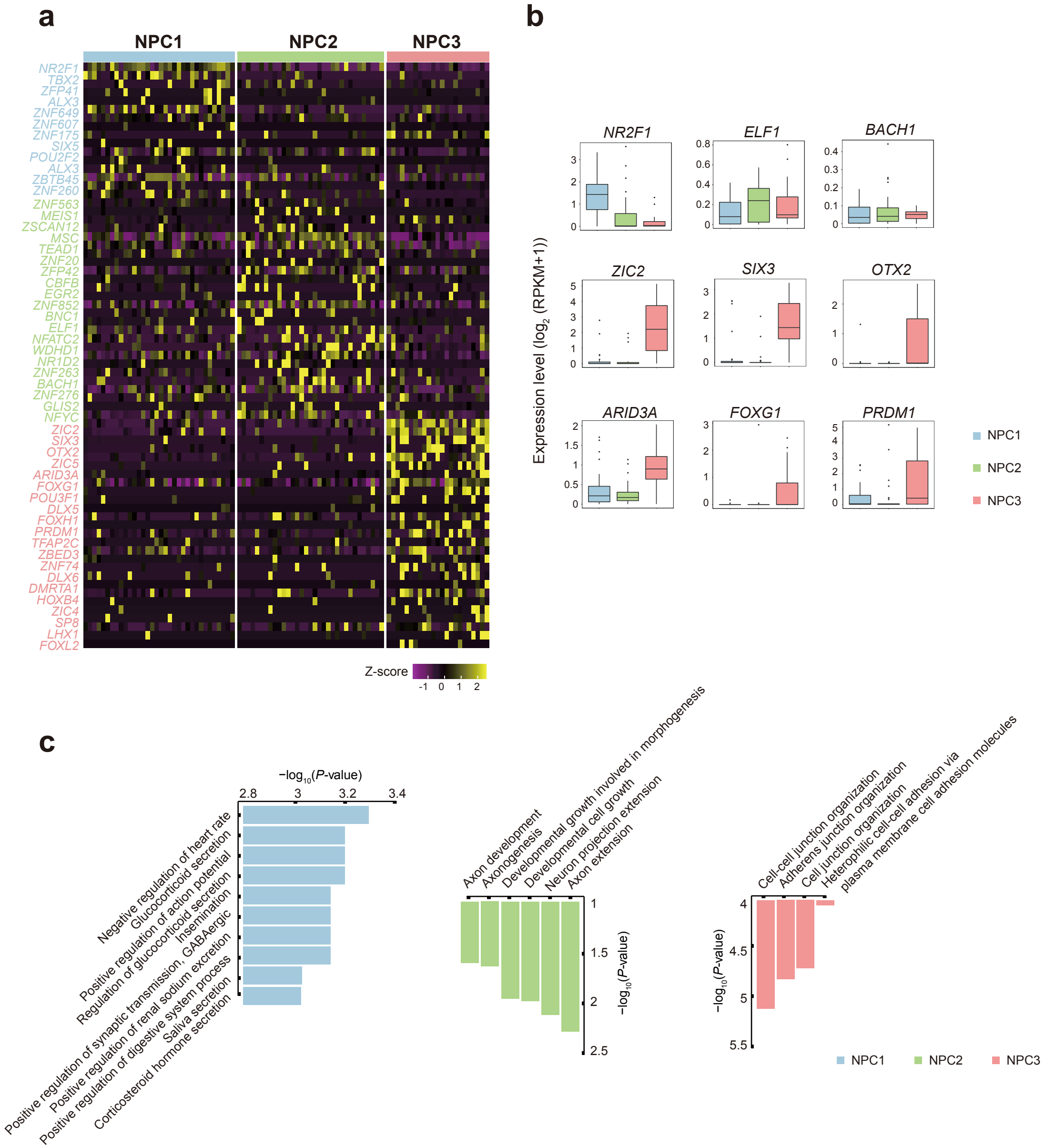

Additional file 8: Figure S8. Subgroups identification and key transcriptomic features within NPCs stage. a Heatmap reports scaled expression [$\log_2$(RPKM+1)] of discriminative TF sets for each cluster in NPCs stage with *P*-value cutoff ≤ 0.01. Color scheme is based on z-score distribution from -1 (purple) to 2 (yellow). Gene symbols highlight with color specific to the respective NPC subset. **b** Box plot of selected TFs defined in Figure S8a. **c** Top 10 (NPC1) and all (NPC2 and NPC3) of GO terms identified by up-regulated genes specific to the respective Ros-L subpopulation with the color as indicated (blue: GO terms specific to NPC1; green: GO terms specific to NPC2; pink: GO terms specific to NPC3).

Additional file 9: Figure S9

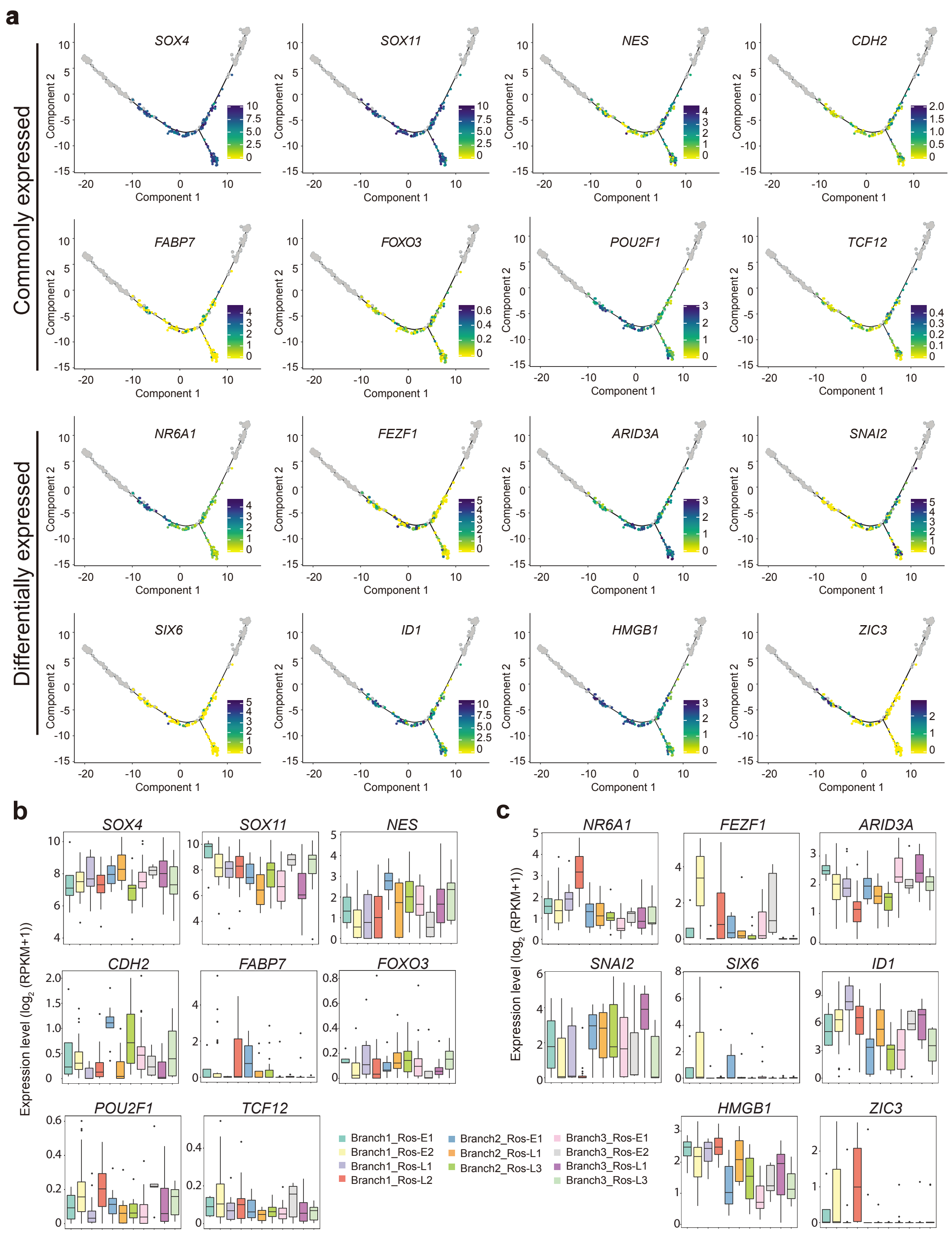

Additional file 9: Figure S9. Expression pattern of selected transcription factors (TFs) within rosettes (Ros-E and Ros-L) stage. a Expression enrichment of commonly and differentially expressed TFs along the differentiation trajectory. Color scheme is based on expression [$\log_2$ (RPKM+1)]. **b, c** Expression pattern of selected TFs with respect to Figure S9a (adjusted *P*-value ≤ 0.01).

Additional file 10: Figure S10

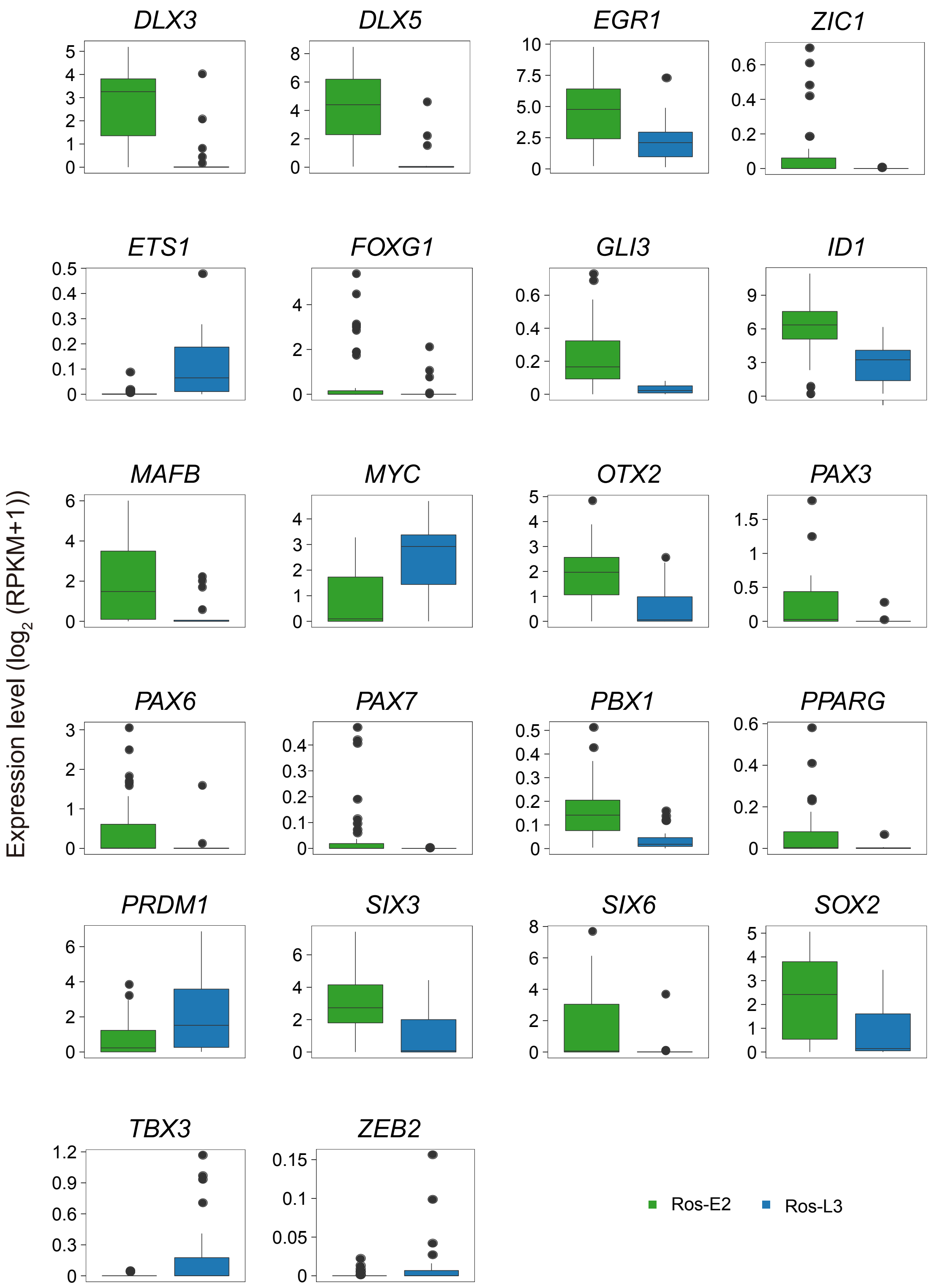

Additional file 10: Figure S10. Differentially expressed transcription factors (TFs) between Ros-E2 and Ros-L3. Ros-E2 and Ros-L3 were shown in green and blue column, respectively (adjusted P-value ≤ 0.01).

Additional file 11: Figure S11

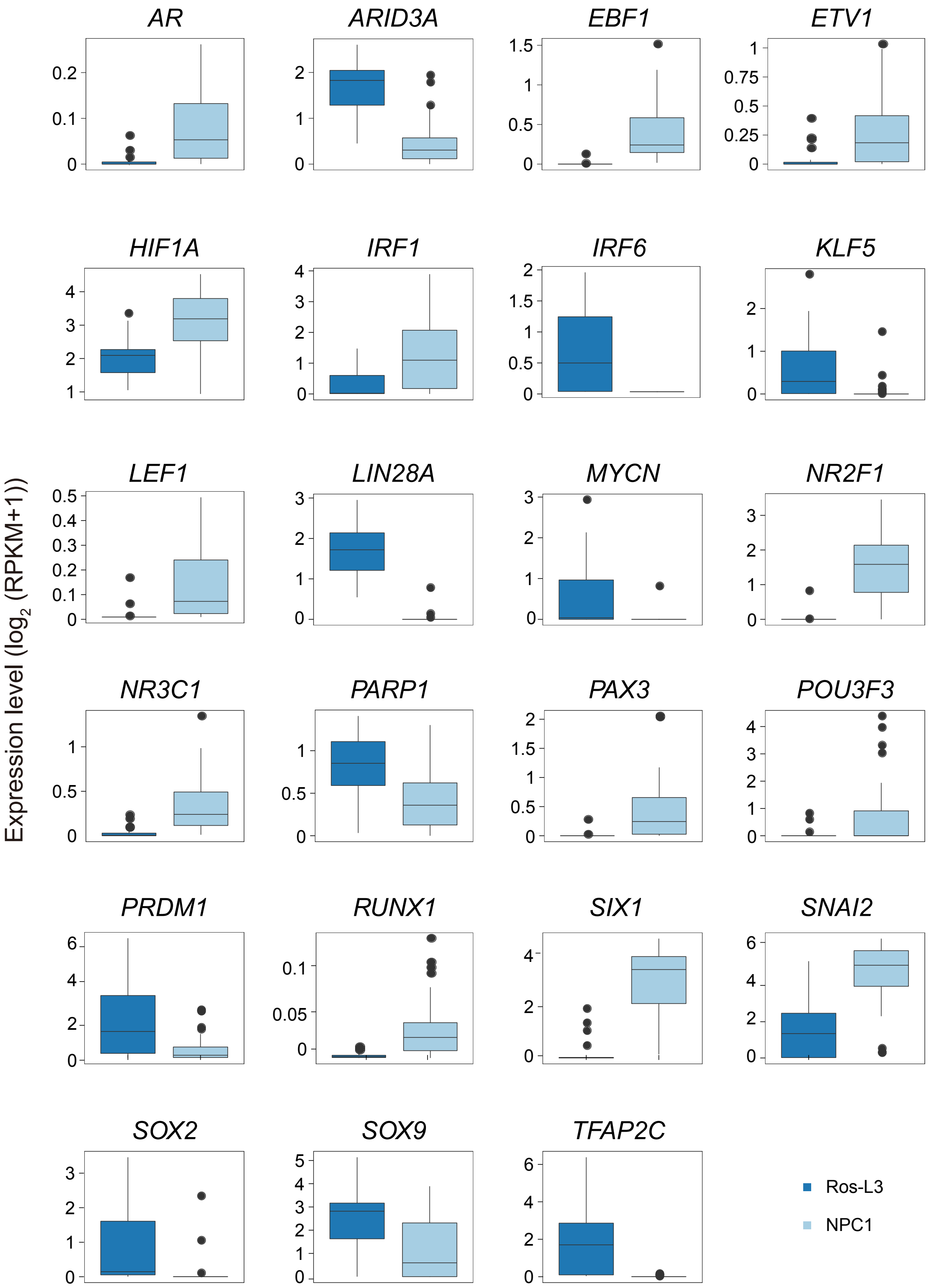

Additional file 11: Figure S11. Differentially expressed transcription factors (TFs) between Ros-L3 and NPC1. Ros-L3 and NPC1 were shown in dark blue and light blue column, respectively (adjusted *P*-value ≤ 0.01).

Additional file 12: Figure S12

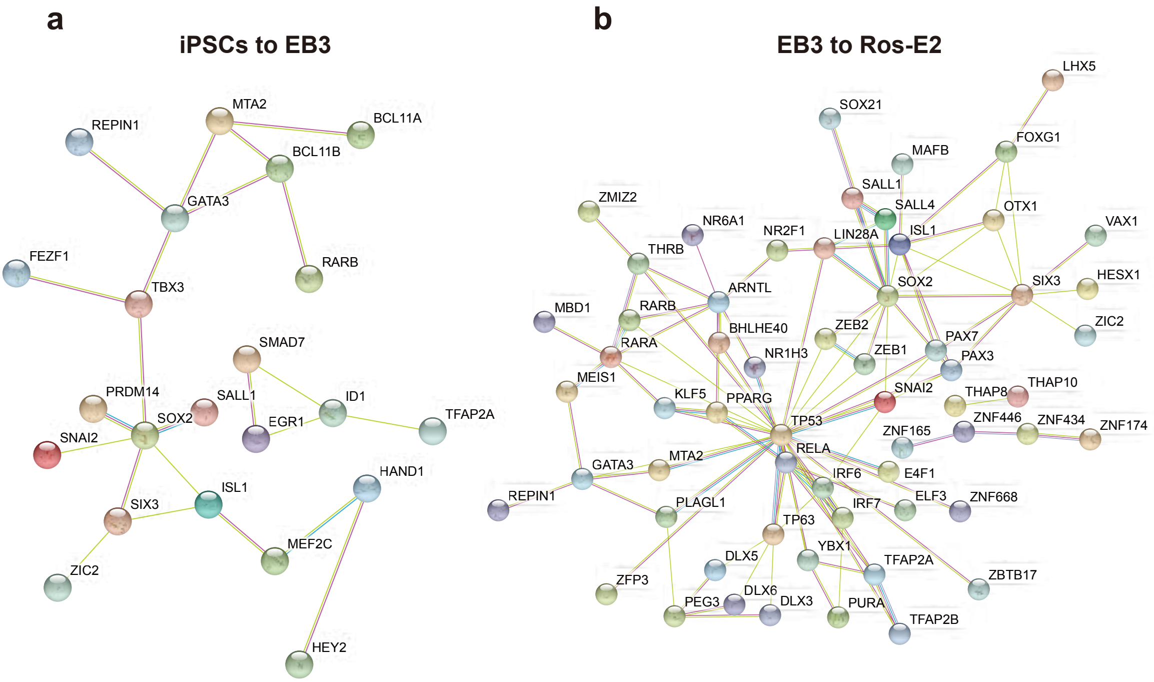

Additional file 12: Figure S12. Key regulators during neural differentiation. a Regulatory network of differentially expressed TFs between iPSCs and EB3. **b** Regulatory network of differentially expressed TFs between EB3 and Ros-E2.

Additional file 13: Figure S13

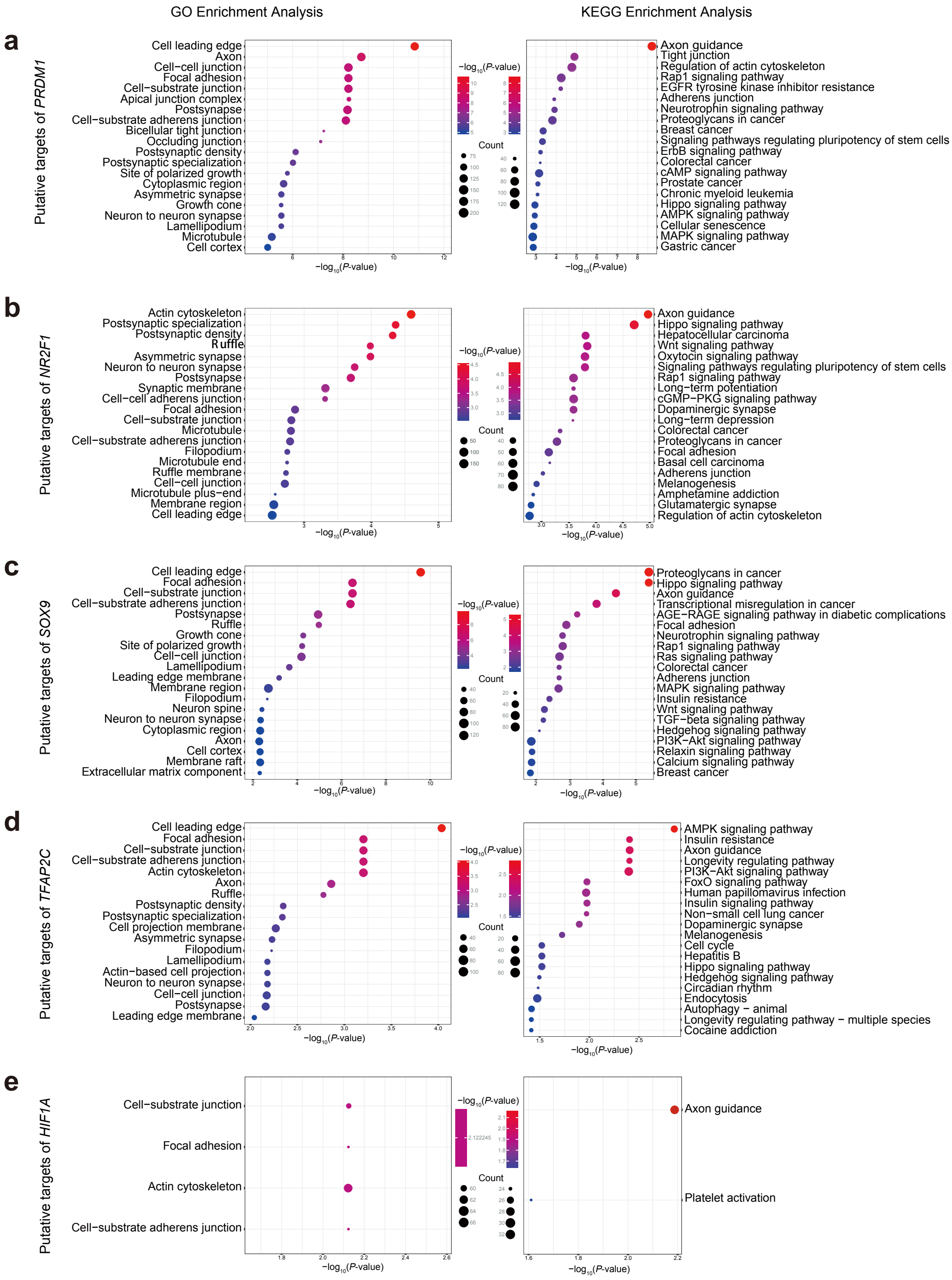

Additional file 13: Figure S13. GO term and KEGG enrichment analysis of selected transcription factors (TFs) targets. GO term and KEGG enrichment analysis for putative targets of *PRDM1* (**a**), *NR2F1* (**b**), *SOX9* (**c**), *TFAP2C* (**d**) and *HIF1A* (**e**), adjusted *P*-value ≤ 0.05.

Additional file 14: Figure S14

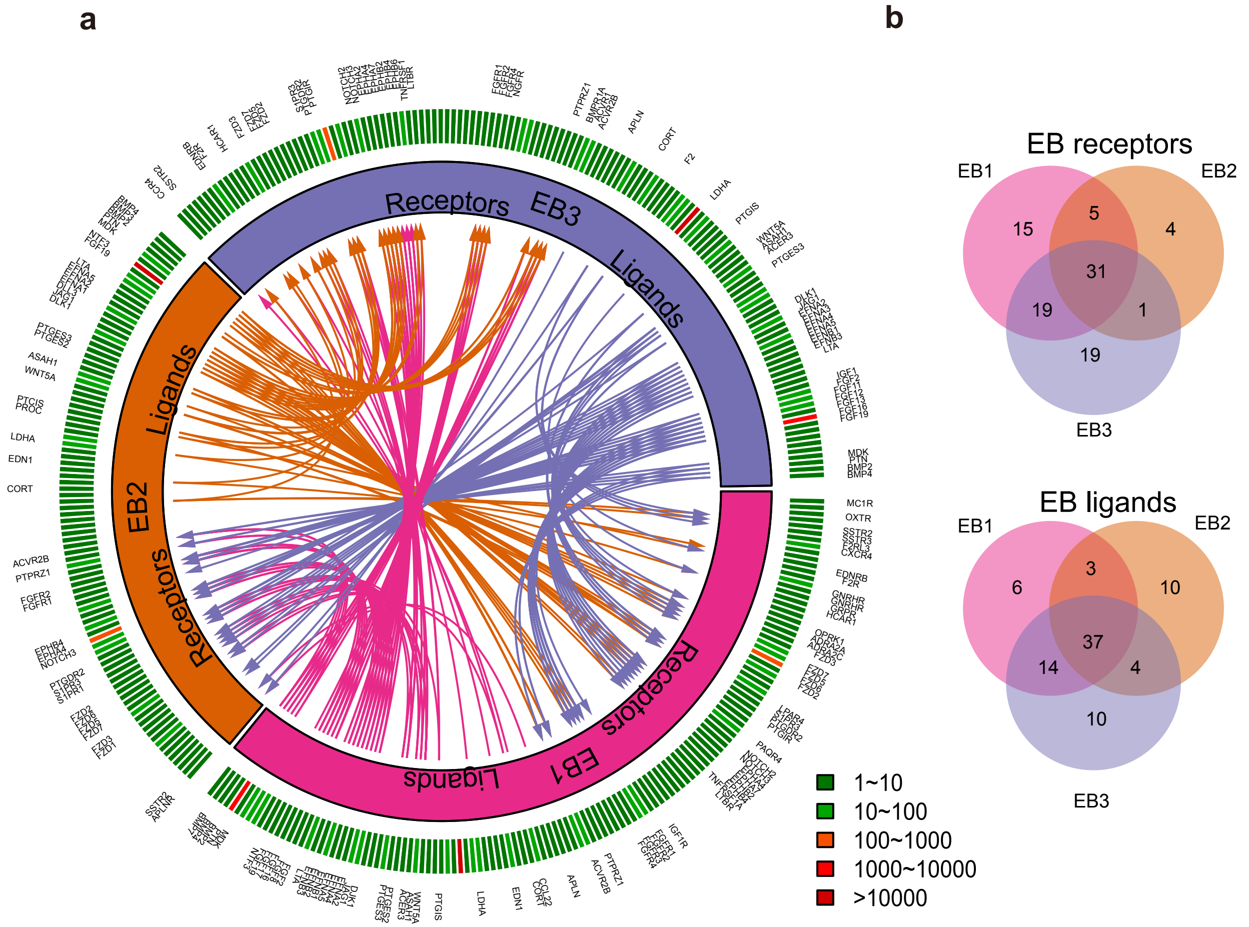

Additional file 14: Figure S14. Putative signaling between expressed receptors and their ligands in EB subsets. a The inner layer compartments represent different cell subpopulations (EB1, EB2 and EB3). The outer layer indicates the expression profiles of ligands and receptors expressed in each cell subset, with low expressed molecular in green color while high expressed ones in red color. Arrows indicate putative interactions between ligands and receptors among cell subsets. **b** Venn plot showing the overlapping of ligands and receptors among EB subpopulations.

Additional file 15: Figure S15

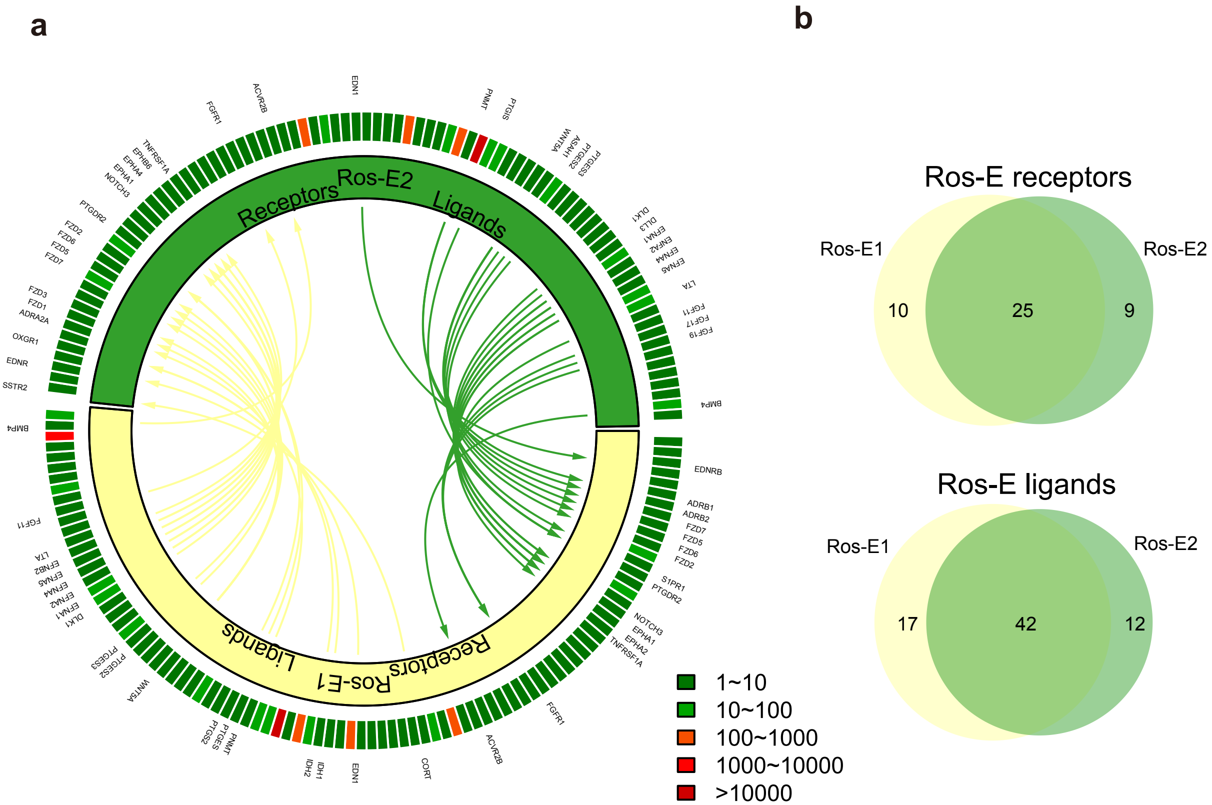

Additional file 15: Figure S15. Putative signaling between expressed receptors and their ligands in Ros-E subsets. a The inner layer compartments represent different cell subpopulations (Ros-E1 and Ros-E2). The outer layer indicates the expression profiles of ligands and receptors expressed in each cell subset, with low expressed molecular in green color while high expressed ones in red color. Arrows indicate putative interactions between ligands and receptors among cell subsets. **b** Venn plot showing the overlapping of ligands and receptors between Ros-E subpopulations.

Additional file 16: Figure S16

a

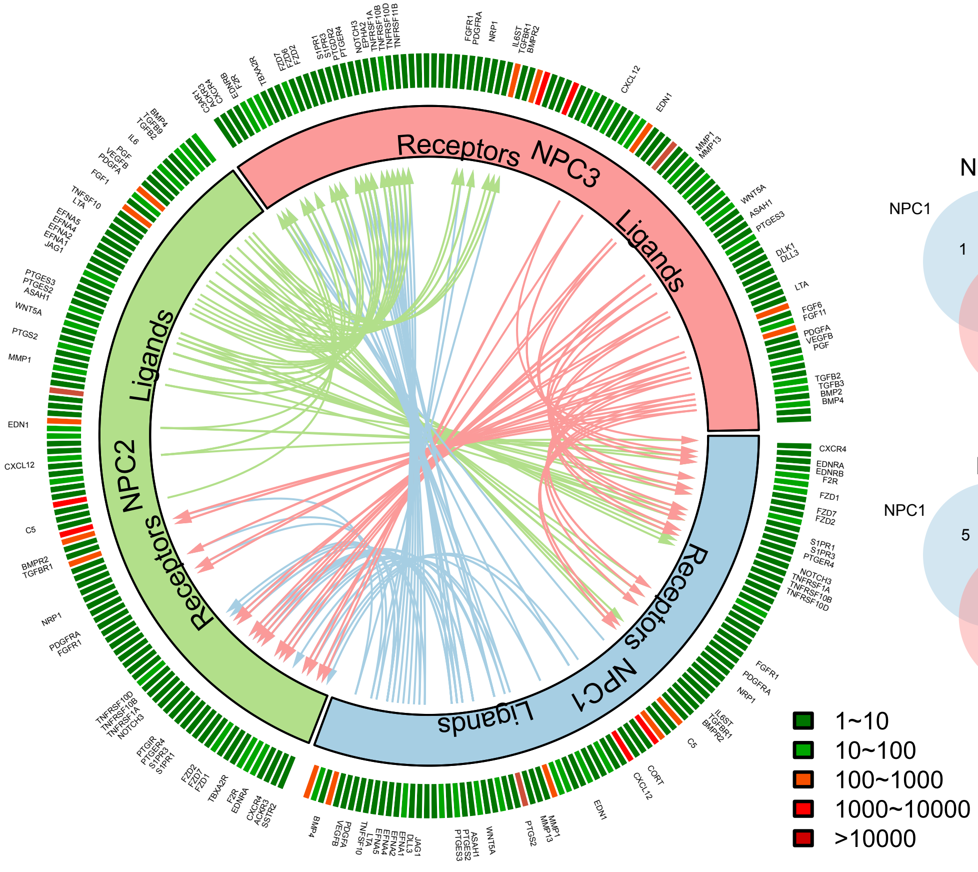

b

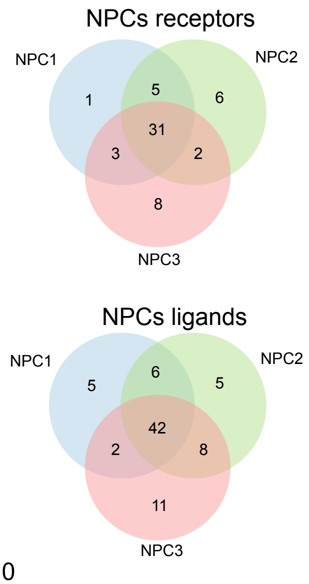

Additional file 16: Figure S16. Putative signaling between expressed receptors and their ligands in NPC subsets. a The inner layer compartments represent different cell subpopulations (NPC1, NPC2 and NPC3). The outer layer indicates the expression profiles of ligands and receptors expressed in each cell subset, with low expressed molecular in green color while high expressed ones in red color. Arrows indicate putative interactions between ligands and receptors among cell subsets. **b** Venn plot showing the overlapping of ligands and receptors among NPC subpopulations.

Additional file 17: Figure S17

a. iPSCs stage

Motif	*P*-value	Best Match/Details
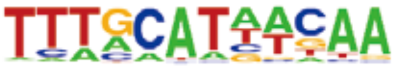	1e-279	Pou5f1::Sox2/MA0142.1/Jaspar(0.924)
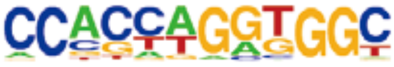	1e-73	BORIS(Zf)/K562-CTCFL-ChIP-Seq(GSE32465)/Homer(0.899)
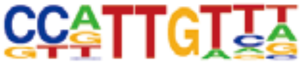	1e-57	Sox3(HMG)/NPC-Sox3-ChIP-Seq(GSE33059)/Homer(0.956)
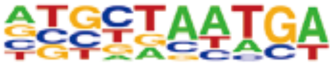	1e-47	Oct6(POU,Homeobox)/NPC-Oct6-ChIP-Seq(GSE35496)/Homer(0.693)
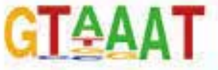	1e-43	FOXB1/MA0845.1/Jaspar(0.759)
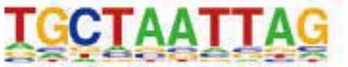	1e-41	PH0098.1_Lhx8/Jaspar(0.881)
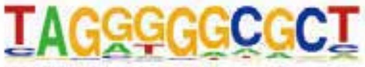	1e-37	BORIS(Zf)/K562-CTCFL-ChIP-Seq(GSE32465)/Homer(0.753)
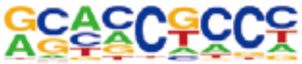	1e-33	POL003.1_GC-box/Jaspar(0.820)
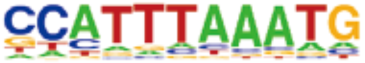	1e-28	LIN54/MA0619.1/Jaspar(0.778)
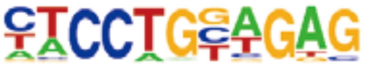	1e-27	Unknown-ESC-element/mES-Nanog-ChIP-Seq(GSE11724)/Homer(0.797)
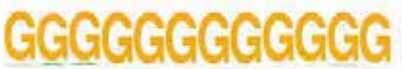	1e-25	PB0097.1_Zfp281_1/Jaspar(0.919)
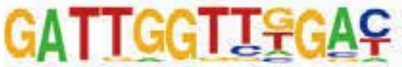	1e-25	NFYB/MA0502.1/Jaspar(0.736)
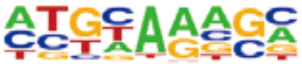	1e-24	MF0006.1_bZIP_cEBP-like_subclass/Jaspar(0.595)
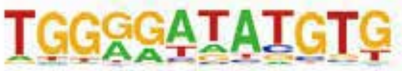	1e-23	MZF1/MA0056.1/Jaspar(0.610)
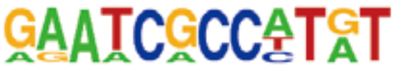	1e-22	YY2/MA0748.1/Jaspar(0.660)

b. EB stage

Motif	*P*-value	Best Match/Details
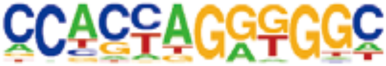	1e-298	BORIS(Zf)/K562-CTCFL-ChIP-Seq(GSE32465)/Homer(0.932)
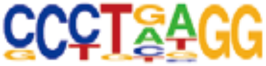	1e-74	AP-2alpha(AP2)/Hela-AP2alpha-ChIP-Seq(GSE31477)/Homer(0.877)
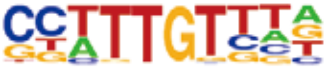	1e-58	Sox3(HMG)/NPC-Sox3-ChIP-Seq(GSE33059)/Homer(0.962)
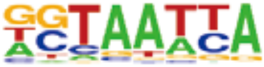	1e-57	MEOX1/MA0661.1/Jaspar(0.902)
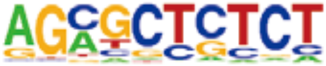	1e-53	PB0099.1_Zfp691_1/Jaspar(0.622)
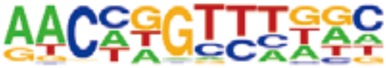	1e-51	GRHL1/MA0647.1/Jaspar(0.859)
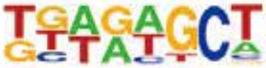	1e-50	POL010.1_DCE_S_III/Jaspar(0.698)
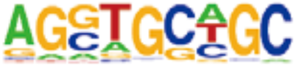	1e-44	PRDM9(Zf)/Testis-DMC1-ChIP-Seq(GSE35498)/Homer(0.685)
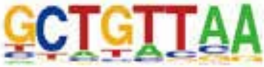	1e-44	Rhox11/MA0629.1/Jaspar(0.782)
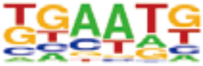	1e-42	TEAD(TEA)/Fibroblast-PU.1-ChIP-Seq(Unpublished)/Homer(0.810)
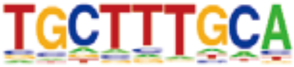	1e-41	POU2F2/MA0507.1/Jaspar(0.671)
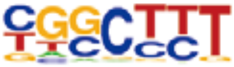	1e-39	Nr5a2(NR)/mES-Nr5a2-ChIP-Seq(GSE19019)/Homer(0.624)
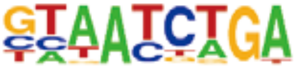	1e-36	Six1(Homeobox)/Myoblast-Six1-ChIP-Chip(GSE20150)/Homer(0.836)
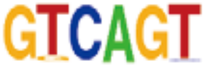	1e-34	Myb/MA0100.2/Jaspar(0.769)
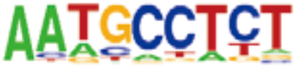	1e-33	PH0137.1_Pitx1/Jaspar(0.653)

c. Ros-E stage

Motif	*P*-value	Best Match/Details
AAAGGAATGT	1e-30	TEAD4/MA0809.1/Jaspar(0.941)
ACACCAGGTGGC	1e-22	CTCF(Zf)/CD4+-CTCF-ChIP-Seq(Barski_et_al.)/Homer(0.833)
CCTAATTA	1e-19	Lhx2(Homeobox)/HFSC-Lhx2-ChIP-Seq(GSE48068)/Homer(0.951)
AACCGGTTT	1e-18	TFCP2/MA0145.3/Jaspar(0.933)
GTTGCCATGGCA	1e-17	Rfx1(HTH)/NPC-H3K4me1-ChIP-Seq(GSE16256)/Homer(0.937)
AAACAATG	1e-16	Sox15(HMG)/CPA-Sox15-ChIP-Seq(GSE62909)/Homer(0.882)
ATAACTCCGCAG	1e-16	Nrf2(bZIP)/Lymphoblast-Nrf2-ChIP-Seq(GSE37589)/Homer(0.772)
GTCAGTCA	1e-15	Pknox1(Homeobox)/ES-Prep1-ChIP-Seq(GSE63282)/Homer(0.853)
CTGCCTGTTGA	1e-15	Ets1-distal(ETS)/CD4+-PolII-ChIP-Seq(Barski_et_al.)/Homer(0.676)
CTGTAAACCACT	1e-15	RUNX1(Runt)/Jurkat-RUNX1-ChIP-Seq(GSE29180)/Homer(0.691)
AAAGGCCTT	1e-14	ZFX(Zf)/mES-Zfx-ChIP-Seq(GSE11431)/Homer(0.790)
TTTATGGGTTAG	1e-14	CRX(Homeobox)/Retina-Crx-ChIP-Seq(GSE20012)/Homer(0.688)
TGACATC	1e-13	MEIS1/MA0498.2/Jaspar(0.906)
TGTATAAGCAGA	1e-13	FoxL2(Forkhead)/Ovary-FoxL2-ChIP-Seq(GSE60858)/Homer(0.610)
TGTTCAGAG	1e-13	AR-halfsite(NR)/LNCaP-AR-ChIP-Seq(GSE27824)/Homer(0.627)

d. Ros-L stage

Motif	*P*-value	Best Match/Details
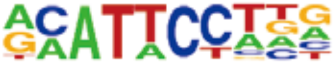	1e-156	TEAD4/MA0809.1/Jaspar(0.970)
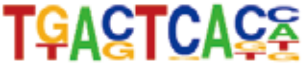	1e-61	FOSL1/MA0477.1/Jaspar(0.944)
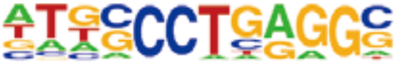	1e-45	AP-2alpha(AP2)/Hela-AP2alpha-ChIP-Seq(GSE31477)/Homer(0.967)
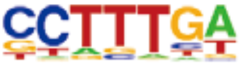	1e-38	Tcf3(HMG)/mES-Tcf3-ChIP-Seq(GSE11724)/Homer(0.891)
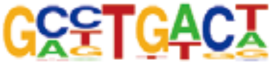	1e-27	Pax2/MA0067.1/Jaspar(0.780)
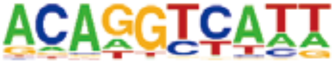	1e-26	Nur77(NR)/K562-NR4A1-ChIP-Seq(GSE31363)/Homer(0.750)
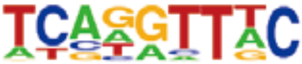	1e-23	Six1(Homeobox)/Myoblast-Six1-ChIP-Chip(GSE20150)/Homer(0.903)
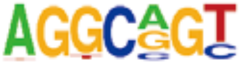	1e-21	MYB(HTH)/ERMYB-Myb-ChIPSeq(GSE22095)/Homer(0.749)
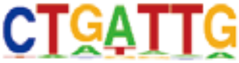	1e-20	Dux/MA0611.1/Jaspar(0.880)
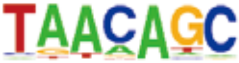	1e-18	Rhox11/MA0629.1/Jaspar(0.851)
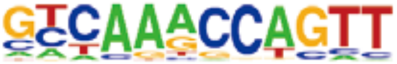	1e-18	TFCP2/MA0145.3/Jaspar(0.756)
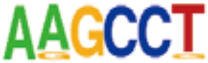	1e-17	ZFX(Zf)/mES-Zfx-ChIP-Seq(GSE11431)/Homer(0.814)
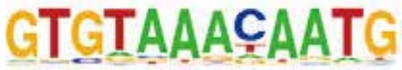	1e-17	FOXO3/MA0157.2/Jaspar(0.748)
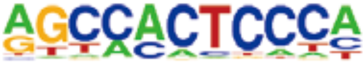	1e-17	KLF5(Zf)/LoVo-KLF5-ChIP-Seq(GSE49402)/Homer(0.804)
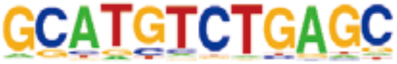	1e-16	Bach1::Mafk/MA0591.1/Jaspar(0.632)

e. NPCs stage

Motif	*P*-value	Best Match/Details
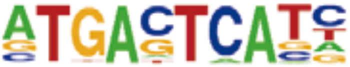	1e-7054	AP-1(bZIP)/ThioMac-PU.1-ChIP-Seq(GSE21512)/Homer(0.990)
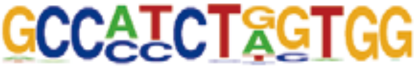	1e-1065	CTCF(Zf)/CD4+-CTCF-ChIP-Seq(Barski_et_al.)/Homer(0.920)
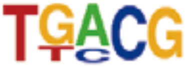	1e-928	Atf1/MA0604.1/Jaspar(0.898)
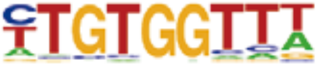	1e-776	RUNX(Runt)/HPC7-Runx1-ChIP-Seq(GSE22178)/Homer(0.989)
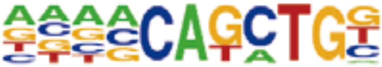	1e-524	Ascl1(bHLH)/NeuralTubes-Ascl1-ChIP-Seq(GSE55840)/Homer(0.961)
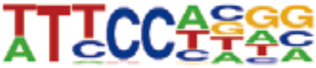	1e-475	NFATC1/MA0624.1/Jaspar(0.870)
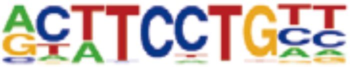	1e-259	Ets1-distal(ETS)/CD4+-PolII-ChIP-Seq(Barski_et_al.)/Homer(0.935)
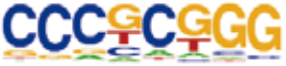	1e-251	TFAP2A(var.2)/MA0810.1/Jaspar(0.780)
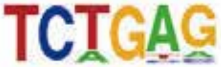	1e-241	Smad4(MAD)/ESC-SMAD4-ChIP-Seq(GSE29422)/Homer(0.652)
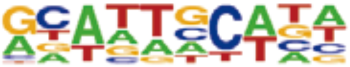	1e-226	TEAD(TEA)/Fibroblast-PU.1-ChIP-Seq(Unpublished)/Homer(0.754)
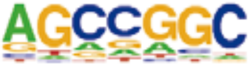	1e-202	POL010.1_DCE_S_III/Jaspar(0.646)
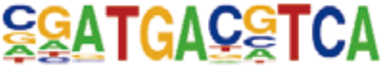	1e-189	c-Jun-CRE(bZIP)/K562-cJun-ChIP-Seq(GSE31477)/Homer(0.958)
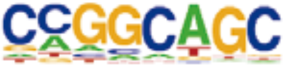	1e-186	POL010.1_DCE_S_III/Jaspar(0.711)
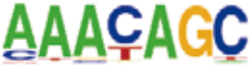	1e-170	Foxo1(Forkhead)/RAW-Foxo1-ChIP-Seq(Fan_et_al.)/Homer(0.767)
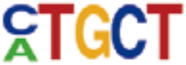	1e-168	POL010.1_DCE_S_III/Jaspar(0.795)

Additional file 17: Figure S17. Transcription factor motifs enriched in stage specific peaks. Motifs enriched in stage specific ATAC peaks were listed (top 15) in tables containing the following information: motif, *P*-value and best match/details for iPSCs (**a**), EB (**b**), Ros-E (**c**), Ros-L (**d**) and NPCs stage (**e**), respectively.

Additional file 18: Figure S18

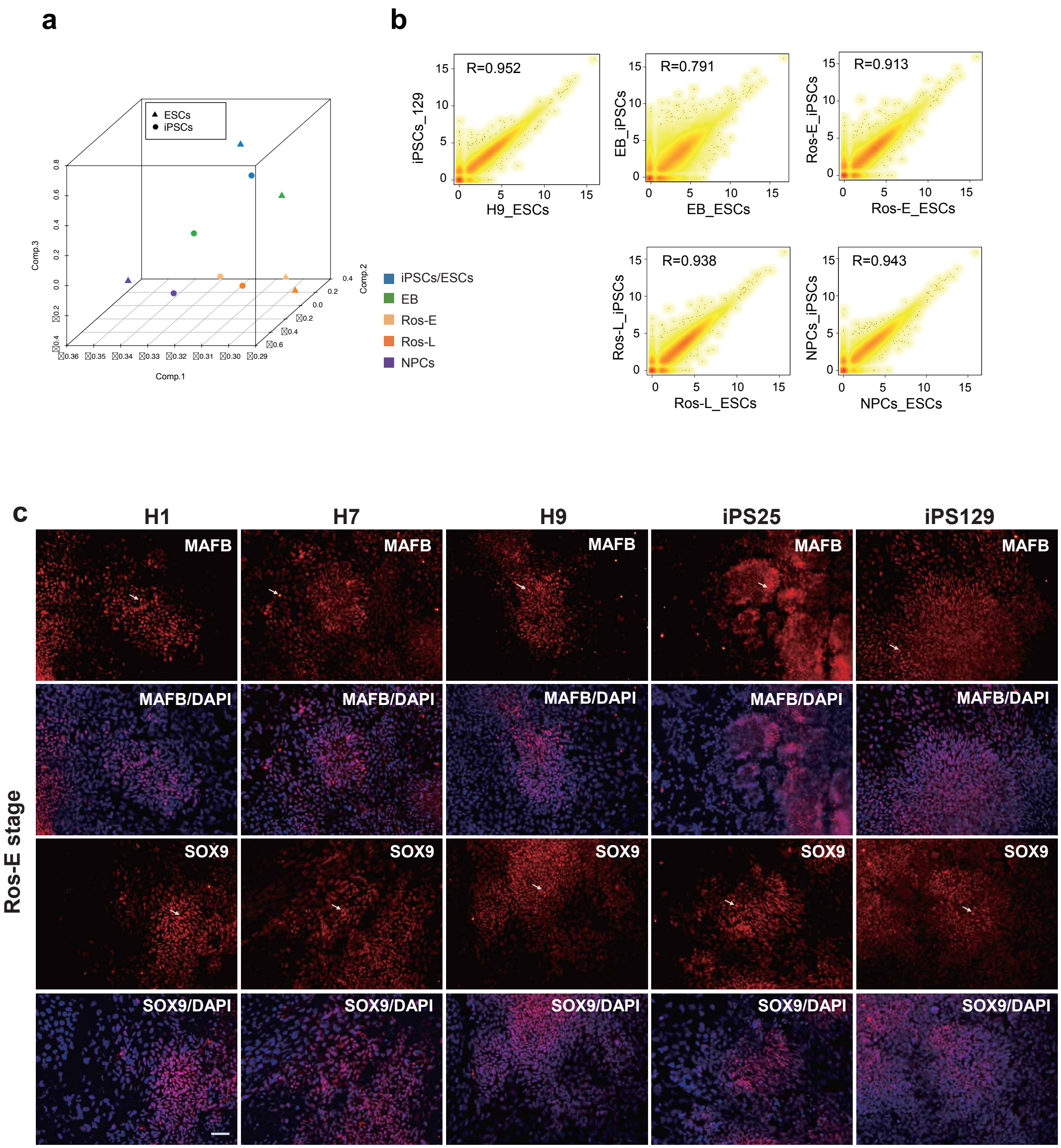

Additional file 18: Figure S18

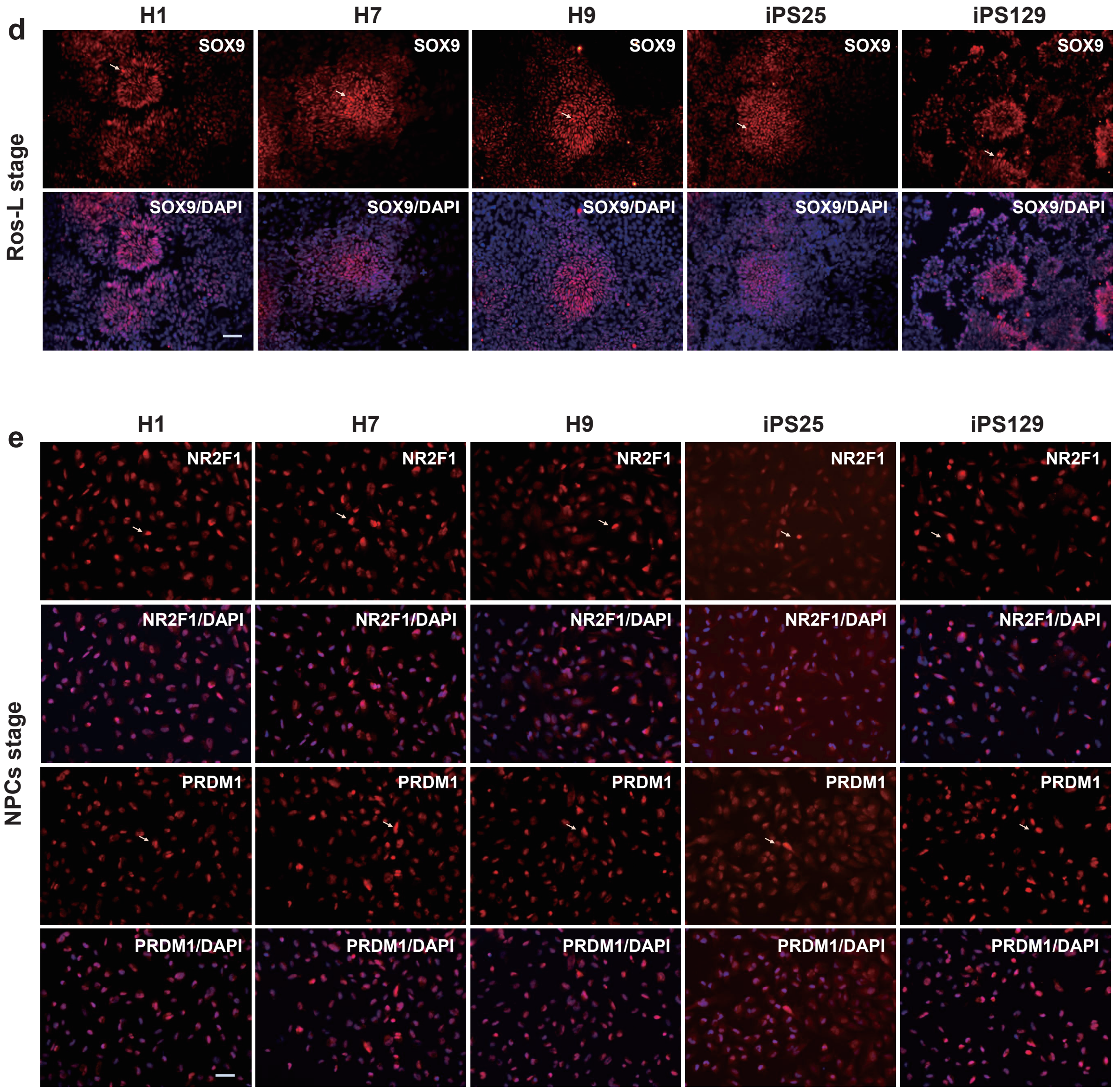

Additional file 18: Figure S18. Validation of neural differentiation in different genetic background cell lines. a 3D PCA plot of the indicated cell stage derived from ESCs or iPSCs designated by colors and symbols. **b** The Pearson correlation coefficient between the corresponding cell stage derived from iPSCs and ESCs. **c, d, e** Immunostaining of MAFB and SOX9 at Ros-E stage (**c**), SOX9 at Ros-L stage (**d**), NR2F1 and PRDM1 at NPCs stage (**e**) across different genetic background cell lines (H1_ESCs, H7_ESCs, H9_ESCs, iPS25 and iPS129). Scale bar represents 50 μm.

Additional file 19: Table S1. Differentially expressed TFs among neighbouring cell subsets.

Additional file 20: Table S2. Putative targets of selected regulators.

Additional file 21: Table S3. Ligand-receptor interaction networks among subpopulations.

Additional file 22: Table S4. Differentially expressed receptors and ligands among Ros-L subpopulations.
