# Supplementary material for: Single-cell RNA-seq reveals dynamic transcriptome profiling in human early neural differentiation
Source: Gigascience. 2018 Sep 18;7(11):giy117. doi: 10.1093/gigascience/giy117 (PMC6420650; doi:10.1093/gigascience/giy117)
Supplement: Authors_Response_To_Reviewer_Comments_Revision_1.pdf [file giy117_authors_response_to_reviewer_comments_revision_1.pdf]

Author's Response To Reviewer Comments

Close

Reviewer reports:
Reviewer #1: The authors did great job addressing all the comments provided. I recommend publication.

Minor point:
The antibodies/dilution used in the revised manuscript should be included in the methods.
Reply: We thank the reviewer for pointing out this incomplete description. Accordingly, we have added the related information in “Materials and Methods-Immunofluorescence staining” (page 29, line 823-835).

Reviewer #2: The authors addressed all of my comments in a satisfying manner, and I recommend the revised manuscript for publication. I wish the authors luck with this interesting paper and their follow up studies.
Reply: We thank the reviewer very much for the insightful and constructive suggestions, which helped us a great deal.

Editor:
(1) The antibodies/dilution used in the revised manuscript should be included in the methods.
Reply: Thanks to the editor, we have added the related information in “Materials and Methods- Immunofluorescence staining” (page 29, line 823-835).

(2) Please ensure that the protocols.io citations under "availability of supporting data" are listed in the references and only the reference number cited in the paper.
Reply: Thanks to the editor, accordingly, we have modified this in the text (Availability of data and materials; page 35, line 994-997).

Close
