# Supplementary material for: Single-cell RNA-seq reveals dynamic transcriptome profiling in human early neural differentiation
Source: Gigascience. 2018 Sep 18;7(11):giy117. doi: 10.1093/gigascience/giy117 (PMC6420650; doi:10.1093/gigascience/giy117)
Supplement: Response_To_Reviewer_Comments_(Original_Submission).pdf [file giy117_response_to_reviewer_comments_(original_submission).pdf]

Author's Response To Reviewer Comments

Close

Reviewer reports:
Reviewer #1: In this study, the authors characterized the single-cell transcriptional profiles as well as chromatin accessibility at several stages during in vitro neuronal differentiation of human iPSCs. Bioinformatics analyses distinguished different subpopulations at each stage and identified transcription factors regulating neural differentiation. Overall, the methods and analyses performed are thoroughly explained, and the experimental results support the author's conclusions.

1- The authors captured single cells of human iPSCs (n = 80), embryoid bodies (EBs; n = 81), early and late rosettes (Ros-E, Ros-L; n = 82 and 93, respectively), NPCs (n = 95), and the original somatic fibroblasts (n = 96). In the manuscript, however, the authors do not show any results of the analysis of the fibroblasts (other than the number of expressed genes in Figure S3d). It is stated that bulk ATAC-seq (for chromatin accessibility) was performed on all these stages, but no data for fibroblasts was shown. If the authors have performed single-cell RNA-seq on somatic fibroblasts as stated in the data description (line 114), this data should be presented in the main or supplementary figures.
Reply: We agree with the reviewer and have now added heterogeneity study of fibroblasts in the revised version (Additional file 5: Figure S5). Considering this study was mainly focused on the regulation of neural differentiation starting from induced pluripotent stem cells (iPSCs), we thus did not include fibroblasts in the ATAC-seq analyses.

2- Figure S2a and Figure S3b/d/e/f are missing y-axis labels. Although it is in the Figure legends, the authors should label the axes directly on the graphs.
Reply: We thank the reviewer for pointing out the mistakes, we totally agree and have accordingly corrected them in the revised version (Additional file 3: Figure S3a and Additional file 4: Figure S4b, d, e, f).

3- Manuscript lines 239-241 and Figure 1f: The authors state that "some important neural transcription factors exhibited heterogeneous expression within the same cell stage (Figure 1f)", but it is difficult to assess this from Figure 1f. I suggest that the authors show evidence for this statement with an updated heatmap or separate analysis that focuses only on these stage-specific genes.
Reply: We agree with the reviewer that the Fig. 1f is not clear enough, thus we modified Figure S4h (Additional file 4) to make it more clearly for showing heterogeneous expression of differentially expressed genes within the same cell stage.

4- Could the authors please double-check that Figure 2c displays the expression levels as log2(RPKM+1), as stated in the legend? The values seem very low. Could it be the z-score instead of RPKM?
Reply: The expression level shown in Fig. 2c is log2 (RPKM +1). All of these genes in Fig. 2c are transcription factors (TFs) coding genes that possess relatively low expression level, which is consistent with the previous publications, e.g., POU5F1, NANOG, SOX2 in Figure 7b; ZIC2, ZIC5 in Figure 7d; KLF4, PRDM14, DPPA2 in Figure 7e (Han et al., 2018); besides, some more published papers with similar gene expression level e.g., Oas2, Lsg20, Lkbke and Tspo in Figure 2 B, F, G, K, L, O, P (Friedman et al., 2018); TH and DCC in Figure 4A (Sousa et al., 2017); Il25 and Tslp in Figure 4c; IL33 in Extended Data Figure 7e;

Retnlb, Wars, Pnliprp2 in Extended Data Figure 10d (Haber et al., 2017). Moreover, in our study, several TFs with relatively low expression levels in scRNA-seq data were validated by immunostaining and showed highly enriched at respective cell stage e.g., SOX9 and MAFB at Ros-E stage, SOX9 and PRDM1 at Ros-L stage, and NR2F1 and PRDM1 at NPCs stage (Fig. 4h; Additional file 18, Figure S18).

5- Figure 2d: It is unclear to me why there are two different -log10(P-value) graphs for the overlapped GO terms of Ros-E1 and Ros-E2 in Figure 2d (in grey color). Same for the overlap in GO terms of Ros-L1 and Ros-L3 in Figure S5. If the authors perform GO enrichment on the list of genes overlapping between the two stages, there should be one set of GO enrichment results?
Reply: We identified enriched GO terms using up-regulated genes for each subpopulation respectively, and analyzed the relationship between the GO terms in different subpopulations within the same cell stage, so the Venn diagram showed the specific GO terms as well as the overlapped GO terms for the indicated subpopulation. Regarding subpopulation specific GO terms, there is only 1 P-value for each term showing their enrichment in corresponding subpopulation. In contrast, regarding those GO terms shared by Ros-E1 and Ros-E2, they are enriched in both subpopulations with different significances. Therefore, each shared GO term have 2 P-values. To display the P-values of each shared GO term, we present the 2 P-values (one from Ros-E1 and another one from Ros-E2) in Figure 2d.

6- Figure 5 c,d,e: The expression levels in log2(RPKM+1) for most of the genes indicated are very low (e.g., NMU: < 0.05; EPHA7, ACKR3, C5, PTPRZ1, ANGPT2: < 0.5). Some of the gene expression levels in other figures (e.g., Figure S4 and Figure S8) are also quite low. Can the authors please explain what log2(RPKM+1) threshold was used for gene detection and/or the filtering out of non-expressed genes?
Reply: The average expression level of RPKM of 1 was used as a threshold. Ligands and receptors above the threshold were considered as expressed in the corresponding subpopulation in figure 5a and 5b. After checking the expression profiles, we figured out the figure was mislabeled in figure 5c, d, e. Specifically, the y axis should be labeled as log10(RPKM+1) instead of log2(RPKM+1). To be consistent with other figures, we have thus regenerated the figure and gene expression level visualized in the form of log2(RPKM+1). In terms of the boxplot, we only kept ligands/receptors with average RPKM>=1 in one subpopulation and average RPKM<1 in other subpopulations. As a result, WNT5A and EPHB6 from Ros-L1, FZD5 and LPAR4 from Ros-L2, ANGPT2 and PGF from Ros-L3 were visualized in figure 5c, d, e, respectively. Regarding Figure S4 and Figure S8 (correspondingly changed to Figure S6 and Figure S10 in the revised version), as mentioned in the reply to question 4, we did not apply a threshold for TFs as they could potentially function at relatively low expression level.

Minor edits:
- Manuscript lines 196-198: incomplete sentence ("Single cells using Smart-Seq2 method [30], followed by sequencing around 6 million reads per cell.)
Reply: Thanks a lot for pointing out this incomplete sentence, it has been updated into "Single cell RNA-seq libraries were generated using Smart-Seq2 method [30], followed by sequencing around 6 million reads per cell."

- I suggest that the authors re-order Figure S4 for better readability (Figure S4d first, then

Figure S4c and finally Figure S4a,b), since the manuscript discusses results from these figures in this particular order.
Reply: Sorry for the confusion. As suggested, we have re-ordered Figure S4 (changed to Figure S6 in the revised version) and cited corresponding figures in the text.

- Figure 4a,b: For readability, I suggest that the authors provide graph titles for the gene networks displayed, to make clear that one is looking at transcription factors differentially expressed between Ros-E2 and Ros-L3 (in Fig. 4a) and between Ros-L3 and NPC-1 (in Fig. 4b).
Reply: Thanks very much for your kind suggestion. We have added graph titles for the gene networks in Fig. 4a, Fig. 4b and Figure S12 following your advice.

- Line 443: redundant et al. - there is a missing reference here, or a reference has been removed.
Reply: Thanks very much for the kind suggestion. We have made the correction in the text.

Reviewer #2: The present manuscript by Shang et al. performed parallel single cell transcriptome and bulk chromatin landscape profiling during six consecutive stages during neural differentiation of fibroblast-based iPSCs. Based on a comprehensive amount of data and detailed analysis, this well-written and well-visualized paper provides many specific and novel insights into gene expression changes specific to neural differentiation. While I feel that this manuscript is a very interesting read as well as a valuable resource for the field, functional validation of cellular heterogeneity, as well as of the putative novel hub TFs would substantially improve the current study. Below please find my specific points that I feel should be addressed prior to publication.

Major points:

1. Heterogeneity
Definition of the subclasses at the different stages is done based on single-cell RNAseq. Based on the presented data, it is not clear if the observed heterogeneity…
i) …stems from the fact that the harvested cells at each stage contain lagging and leading cells (slow and fast differentiating cells).
ii) … represent temporal transcriptional states (e.g. during cell cycle or circadian rhythm).
iii) … represent distinct cellular subpopulation that occur in parallel, but that have different fates (e.g. dorsal, ventral, neural crest, …).
While the Monocle analysis appears to suggest iii), the data is not able to convincingly draw a conclusion in this regard. I thus suggest that the authors should mention this as a caveat more prominently. Alternatively, single-cell ATACseq might help to gain insights if the observed subclasses are also distinguishable epigenetically, but the effort seems immense.
Reply: Thanks to the reviewer for the constructive suggestions. The reviewer has raised a very important point that the current scRNA-seq method by its nature only provides a snapshot of the gene expression profile for individual cells. We have added a discussion part regarding the concerns on the heterogeneity study in the manuscript. In spite of the very interesting heterogeneity and cell fate commitment study inferred above, we cannot exclude the following factors that may affect cell subset identification in the above description; 1) temporal transcriptional states during transient differentiation process; 2) differentiation efficiency; and lagging and leading cells remaining in the differentiation process. However, we propose that the subsets dissection analysis facilitates a more precise description of the

factors defining the differentiation trajectory. When we constructed the differentiation trajectory using the cells that collected at different time points, the results showed that all subpopulations in stages from iPSCs to NPCs followed a sequential differentiation process where each stage exhibited a relatively discriminative region with some of the subpopulations overlapping (Fig. 3a), indicating that in spite of the above concerns, the trajectory was established by the natural features of the respective subsets and which is also supported by the observations that Ros-L2 possessing many early neural differentiation TFs, such as SOX2, OTX2, PAX6, OTX1, and LHX5, as well as forebrain markers (e.g., HESX1) and pluripotency-related TFs (NANOG, SALL4, PRDM14) (Additional file 7： Figure S7) were located in the reconstructed trajectory prior to the generation of Ros-E populations. In addition, we carried out the cell fate commitment analysis using Branch1, Branch2 and Branch3 which were grouped based on the cell locations on the trajectory rather than cell subsets identified by Seurat in order to minimize the above concerns.

2. Validation of heterogeneity
For EBs, Ros-E and Ros-L, the authors find strikingly different subclasses of cells. Based on a few selected 'novel' markers, it would be very interesting to see if this heterogeneity can be confirmed by immunostaining with reasonable effort.
Reply: We agree with the reviewer and we have validated several subset-specific markers experimentally (Fig. 4h; Additional file 18: Figure S18). Briefly, Ros-E (SOX9 and MAFB), Ros-L (SOX9 and PRDM1) and NPCs stage (NR2F1 and PRDM1) were validated by immunostaining, as expected, these TFs showed heterogeneous expression level within the same cell stage, moreover, we also validated these TFs in other ESCs and iPSCs e.g., H1_ESCs, H7_ESCs, H9_ESCs and iPS25, the results were in line with that in iPS129.

3. Fibroblasts
The study analyzed fibroblasts as well, but in most of the presented the analyses they are not included. It would be interesting to see differences relative to fibroblasts also in the Monocle and ATACseq analysis. Also, why are some fibroblasts clustering with EBs in t-SNE (Fig 2a), and how would a PCA of these data look like?
Reply: We agree with the reviewer and we have added heterogeneity study of fibroblasts (Additional file 5: Figure S5), by applying the same subsets identification method. We identified two subsets of fibroblasts, Fib1 and Fib2, and the results showed significantly higher expression of several important pluripotency- and neural-associated transcription factors e.g., SOX2, LIN28, SOX11, ZIC2, FEZF1 and SIX3 in Fib2 (Additional file 5: Figure S5b). We further analyzed the relationship between fibroblast subsets and EBs and we observed that the majority of cells in Fib2 were clustered together with EB cells (Additional file 5: Figure S5e). The same input as Fig. 2a in PCA was shown as below, which recapitulates the cell stage distribution in Fig. 2a. Together with the molecular features of Fib2 subset (Additional file 5: Figure S5b), we proposed that the Fib2 subset might possess high potential for iPSCs reprogramming and neural conversion. Because the neural differentiation started from iPSCs, and we are focusing on studying the regulation of neural differentiation process, we did not include fibroblasts in the trajectory and ATAC-seq analyses.

4. Universal validity / N of genetic background

As that the authors apparently only used one fibroblast line/genetic background for this study, the cogency of the study is limited. While I agree that repeating all experiments with a second line would be very time and money consuming, this caveat leaves the possibility for each finding to be an artifact of this one cell line/genetic background. The authors should try to at least validate some of their key findings/TFs on one another genetic background.
Reply: We understand the concerns raised by the reviewer. To address this, we have performed neural differentiation using ESC with the same protocol and captured bulk transcriptome profiles of the corresponding differentiating cell stages (ESCs, EB, Ros-E, Ros-L and NPCs). The observations in ESCs recapitulated those seen in iPSCs, e.g., 1) PCA analysis; 2) with a high Pearson correlation coefficient between the corresponding cell stage derived from iPSCs and ESCs; 3) validation analysis of subset- specific markers (MAFB, SOX9, PRDM1 and NR2F1) as well as novel neural TF (PRDM1) expression in different genetic cell lines (H1_ESCs, H7_ESCs, H9_ESCs, iPS25 and iPS129) showing consistent with the above heterogeneity study (Fig.4h; Additional file 18: Figure S18).

Regarding the single-cell level, we understand this limitation and are happy to describe it in the discussion because we agree that repeating the entire study with a second line would indeed be very time and money consuming. However, the results inferred from this single line are largely consistent with previously reported findings from a variety of bulk cell-based studies so we feel that the possibility of our transcriptome profiling results being artefactual is extremely low. Our novel findings comprise new information garnered from high-resolution single-cell sequencing, not from experiments that suggest a complete revision of our fundamental understanding of neural differentiation.

5. Functional validation of new TFs
Based on the identification of 'novel' TFs involved in neural differentiation, it would be interesting if overexpression or knockdown of these factors boost/impair neuronal differentiation of iPSCs.
Reply: We have added validation of subset- specific markers as well as novel markers expression in different genetic cell lines (H1_ESCs, H7_ESCs, H9_ESCs, iPS25 and iPS129). As expected, we observed that MAFB, SOX9, PRDM1, NR2F1 were enriched at respective cell stage across different genetic cell lines, and the immunostaining results were consistent with the heterogeneity study. However, additional experiments to validate our novel findings are more appropriate to follow-up studies that can investigate stage-specific regulatory dynamics in more depth.

6. Chromatin closing
For the ATACseq data, in addition to reporting the % novel peaks for each stage, I would be curious to know the % change in peak diversity between each time, because this would take into account both regions of the genome opening up, and regions of the genome closing up, instead of just opening.
Reply: We agree with the reviewer that only analyzing novel peaks might not reflect all the entire chromatin landscape during differentiation stage transitions. As suggested, we included the analysis of gained and lost peaks at each stage and added annotations, especially on the dynamics of lost peak regions (Additional file 2: Figure S2). Briefly, to reveal the detail of chromatin accessibility dynamics during neural differentiation, we analyzed the gained or lost peaks at each stage compared with the previously neighboring one. We observed that the number of gained peaks was with the largest increase at the NPCs stage while the number of lost peaks was relatively high at Ros-E stage (Additional file 2:

Figure S2a). Next, we studied the genomic distribution of these dynamic peaks and found that both the gained and lost peaks were located mostly in distal intergenic regions and promoter regions (Additional file 2: Figure S2b). This observation indicates that distal and promoter regions are more dynamic compared to other genomic regions during neural differentiation process.

To gain insight into the potential function of closing (lost) peaks dynamics, we carried out GO enrichment analysis on the genes annotated by lost peaks at each stage. The GO terms analysis showed that "mesoderm morphogenesis", "endoderm development", "gastrulation" and "nodal signalling pathway" were solely enriched at EB stage, indicating that upstream, as well as other lineage development, was relatively repressed by closing related cis-regulatory regions. Other cell fate conversion terms such as "neural crest cell differentiation", "osteoclast differentiation", and "regulation of cartilage development" were enriched at Ros-E stage, together with the annotation results of novel peaks, indicating that the chromatin accessibility prepared for the neural lineage conversion by opening/closing up specific cis-regulatory regions which facilitated the neural transition cascades (Fig. 1d, e and Additional file 2: Figure S2d, e).

7. Neurons

It would be interesting to see differentiated neurons included in this already very interesting paper!

Reply: Yes, we agree that it would be useful if neurons are included in our study, however, we are afraid that this is probably beyond the scope of this study. This paper mainly focused on early neural differentiation process for following reasons: firstly, early neural development regulatory mechanism remains elusive due to the limited accessibility of human abortive fetuses at such an early stage (week 3 and 4 of human gestation); secondly, another recently published paper has already investigated the differentiation process from neural progenitor cells to neurons (Wang et al., 2017).

Reference

[1] Han X, Chen H, Huang D, Chen H, Fei L, Cheng C, et al. Mapping human pluripotent stem cell differentiation pathways using high throughput single-cell RNA-sequencing. Genome Biol. 2018;19:47.

[2] Friedman BA, Srinivasan K, Ayalon G, Meilandt WJ, Lin H, Huntley MA, et al. Diverse Brain Myeloid Expression Profiles Reveal Distinct Microglial Activation States and Aspects of Alzheimer's Disease Not Evident in Mouse Models. Cell Rep. 2018;22:832-847.

[3] Sousa AMM, Zhu Y, Raghanti MA, Kitchen RR, Onorati M, Tebbenkamp ATN, et al. Molecular and cellular reorganization of neural circuits in the human lineage. Science. 2017;358:1027-1032.

[4] Haber AL, Biton M, Rogel N, Herbst RH, Shekhar K, Smillie C, et al. A single-cell survey of the small intestinal epithelium. Nature. 2017;551:333-339.

[5] Wang J, Jenjaroenpun P, Bhinge A, Angarica VE, Del Sol A, Nookaew I, et al. Single-cell gene expression analysis reveals regulators of distinct cell subpopulations among developing human neurons. Genome Res. 2017;27:1783-1794.

Close
