# Supplementary material for: Single-cell RNA-seq reveals dynamic transcriptome profiling in human early neural differentiation
Source: Gigascience. 2018 Sep 18;7(11):giy117. doi: 10.1093/gigascience/giy117 (PMC6420650; doi:10.1093/gigascience/giy117)
Supplement: Reviewer_1_Report_(Original_Submission) -- Cedric Bardy — 4/23/2018 Reviewed [file giy117_reviewer_1_report_(original_submission).pdf]

Reviewer Report

Title: **Single-cell RNA-seq reveals dynamic transcriptome profiling in human early neural differentiation**

Version: **Original Submission** **Date:** 4/23/2018

Reviewer name: **Cedric Bardy**

Reviewer Comments to Author:

In this study, the authors characterized the single-cell transcriptional profiles as well as chromatin accessibility at several stages during in vitro neuronal differentiation of human iPSCs. Bioinformatics analyses distinguished different subpopulations at each stage and identified transcription factors regulating neural differentiation. Overall, the methods and analyses performed are thoroughly explained, and the experimental results support the author's conclusions.1- The authors captured single cells of human iPSCs (n = 80), embryoid bodies (EBs; n = 81), early and late rosettes (Ros-E, Ros-L; n = 82 and 93, respectively), NPCs (n = 95), and the original somatic fibroblasts (n = 96). In the manuscript, however, the authors do not show any results of the analysis of the fibroblasts (other than the number of expressed genes in Figure S3d). It is stated that bulk ATAC-seq (for chromatin accessibility) was performed on all these stages, but no data for fibroblasts was shown. If the authors have performed single-cell RNA-seq on somatic fibroblasts as stated in the data description (line 114), this data should be presented in the main or supplementary figures.2- Figure S2a and Figure S3b/d/e/f are missing y-axis labels. Although it is in the Figure legends, the authors should label the axes directly on the graphs.3- Manuscript lines 239-241 and Figure 1f: The authors state that "some important neural transcription factors exhibited heterogeneous expression within the same cell stage (Figure 1f)", but it is difficult to assess this from Figure 1f. I suggest that the authors show evidence for this statement with an updated heatmap or separate analysis that focuses only on these stage-specific genes.4- Could the authors please double-check that Figure 2c displays the expression levels as log2(RPKM+1), as stated in the legend? The values seem very low. Could it be the z-score instead of RPKM?5- Figure 2d: It is unclear to me why there are two different -log10(P-value) graphs for the overlapped GO terms of Ros-E1 and Ros-E2 in Figure 2d (in grey color). Same for the overlap in GO terms of Ros-L1 and Ros-L3 in Figure S5. If the authors perform GO enrichment on the list of genes overlapping between the two stages, there should be one set of GO enrichment results?6- Figure 5 c,d,e: The expression levels in log2(RPKM+1) for most of the genes indicated are very low (e.g., NMU: < 0.05; EPHA7, ACKR3, C5, PTPRZ1, ANGPT2: < 0.5). Some of the gene expression levels in other figures (e.g., Figure S4 and Figure S8) are also quite low. Can the authors please explain what log2(RPKM+1) threshold was used for gene detection and/or the filtering out of non-expressed genes?Minor edits:- Manuscript lines 196-198: incomplete sentence ("Single cells using Smart-Seq2 method [30], followed by sequencing around 6 million reads per cell.)- I suggest that the authors re-order Figure S4 for better readability (Figure S4d first, then Figure S4c and finally Figure S4a,b), since the manuscript discusses results from these figures in this particular order.- Figure 4a,b: For readability, I suggest that the authors provide graph titles for the gene networks displayed, to make clear that one is looking at transcription factors differentially expressed between Ros-E2 and Ros-L3 (in Fig. 4a) and between Ros-L3 and NPC-1 (in Fig. 4b).- Line 443: redundant et al. - there is a missing reference here, or a reference has been removed.

Level of Interest

Please indicate how interesting you found the manuscript: Choose an item.

Quality of Written English

Please indicate the quality of language in the manuscript: Choose an item.

Declaration of Competing Interests

Please complete a declaration of competing interests, considering the following questions:

- Have you in the past five years received reimbursements, fees, funding, or salary from an organisation that may in any way gain or lose financially from the publication of this manuscript, either now or in the future?
- Do you hold any stocks or shares in an organisation that may in any way gain or lose financially from the publication of this manuscript, either now or in the future?
- Do you hold or are you currently applying for any patents relating to the content of the manuscript?
- Have you received reimbursements, fees, funding, or salary from an organization that holds or has applied for patents relating to the content of the manuscript?
- Do you have any other financial competing interests?
- Do you have any non-financial competing interests in relation to this paper?

If you can answer no to all of the above, write 'I declare that I have no competing interests' below. If your reply is yes to any, please give details below.

no competing interests.

I agree to the open peer review policy of the journal. I understand that my name will be included on my report to the authors and, if the manuscript is accepted for publication, my named report including any attachments I upload will be posted on the website along with the authors' responses. I agree for my report to be made available under an Open Access Creative Commons CC-BY license (http://creativecommons.org/licenses/by/4.0/). I understand that any comments which I do not wish to be included in my named report can be included as confidential comments to the editors, which will not be published.

I agree to the open peer review policy of the journal

To further support our reviewers, we have joined with Publons, where you can gain additional credit to further highlight your hard work (see: https://publons.com/journal/530/gigascience). On publication of this paper, your review will be automatically added to Publons, you can then choose whether or not to claim your Publons credit. I understand this statement. Yes
