# Supplementary material for: Single-cell RNA-seq reveals dynamic transcriptome profiling in human early neural differentiation
Source: Gigascience. 2018 Sep 18;7(11):giy117. doi: 10.1093/gigascience/giy117 (PMC6420650; doi:10.1093/gigascience/giy117)
Supplement: Reviewer_2_Report_(Original_Submission) -- Jerome Mertens — 4/27/2018 Reviewed [file giy117_reviewer_2_report_(original_submission).pdf]

Reviewer Report

Title: Single-cell RNA-seq reveals dynamic transcriptome profiling in human early neural differentiation

Version: Original Submission **Date:** 4/27/2018

Reviewer name: Jerome Mertens

Reviewer Comments to Author:

The present manuscript by Shang et al. performed parallel single cell transcriptome and bulk chromatin landscape profiling during six consecutive stages during neural differentiation of fibroblast-based iPSCs. Based on a comprehensive amount of data and detailed analysis, this well-written and well-visualized paper provides many specific and novel insights into gene expression changes specific to neural differentiation. While I feel that this manuscript is a very interesting read as well as a valuable resource for the field, functional validation of cellular heterogeneity, as well as of the putative novel hub TFs would substantially improve the current study. Below please find my specific points that I feel should be addressed prior to publication.

Major points:

1. Heterogeneity
Definition of the subclasses at the different stages is done based on single-cell RNAseq. Based on the presented data, it is not clear if the observed heterogeneity…
i) …stems from the fact that the harvested cells at each stage contain lagging and leading cells (slow and fast differentiating cells).
ii) … represent temporal transcriptional states (e.g. during cell cycle or circadian rhythm).
iii) … represent distinct cellular subpopulation that occur in parallel, but that have different fates (e.g. dorsal, ventral, neural crest, …).
While the Monocle analysis appears to suggest iii), the data is not able to convincingly draw a conclusion in this regard. I thus suggest that the authors should mention this as a caveat more prominently. Alternatively, single-cell ATACseq might help to gain insights if the observed subclasses are also distinguishable epigenetically, but the effort seems immense.

2. Validation of heterogeneity
For EBs, Ros-E and Ros-L, the authors find strikingly different subclasses of cells. Based on a few selected 'novel' markers, it would be very interesting to see if this heterogeneity can be confirmed by immunostaining with reasonable effort.

3. Fibroblasts
The study analyzed fibroblasts as well, but in most of the presented the analyses they are not included. It would be interesting to see differences relative to fibroblasts also in the Monocle and ATACseq analysis. Also, why are some fibroblasts clustering with EBs in t-SNE (Fig 2a), and how would a PCA of these data look like?

4. Universal validity / N of genetic background
As that the authors apparently only used one fibroblast line/genetic background for this study, the cogency of the study is limited. While I agree that repeating all experiments with a second line would be very time and money consuming, this caveat leaves the possibility for each finding to be an artifact of this one cell line/genetic background. The authors should try to at least validate some of their key findings/TFs on one another genetic background.

5. Functional validation of new TFs
Based on the identification of 'novel' TFs involved in neural differentiation, it would be interesting if overexpression or knockdown of these factors boost/impair neuronal differentiation of iPSCs.

6. Chromatin closing
For the ATACseq data, in addition to reporting the % novel peaks for each stage, I would be curious to know the % change in peak diversity between each time, because this would take into account both regions of the genome opening up, and regions of the genome closing up, instead of just opening.

7. Neurons
It would be interesting to see differentiated neurons included in this already very interesting paper!

Level of Interest

Please indicate how interesting you found the manuscript: Choose an item.

Quality of Written English

Please indicate the quality of language in the manuscript: Choose an item.

Declaration of Competing Interests

Please complete a declaration of competing interests, considering the following questions:

- Have you in the past five years received reimbursements, fees, funding, or salary from an organisation that may in any way gain or lose financially from the publication of this manuscript, either now or in the future?
- Do you hold any stocks or shares in an organisation that may in any way gain or lose financially from the publication of this manuscript, either now or in the future?
- Do you hold or are you currently applying for any patents relating to the content of the manuscript?
- Have you received reimbursements, fees, funding, or salary from an organization that holds or has applied for patents relating to the content of the manuscript?
- Do you have any other financial competing interests?
- Do you have any non-financial competing interests in relation to this paper?

If you can answer no to all of the above, write 'I declare that I have no competing interests' below. If your reply is yes to any, please give details below.

I declare that I have no competing interests

I agree to the open peer review policy of the journal. I understand that my name will be included on my report to the authors and, if the manuscript is accepted for publication, my named report including any attachments I upload will be posted on the website along with the authors' responses. I agree for my report to be made available under an Open Access Creative Commons CC-BY license

(http://creativecommons.org/licenses/by/4.0/). I understand that any comments which I do not wish to be included in my named report can be included as confidential comments to the editors, which will not be published.

I agree to the open peer review policy of the journal

To further support our reviewers, we have joined with Publons, where you can gain additional credit to further highlight your hard work (see: https://publons.com/journal/530/gigascience). On publication of this paper, your review will be automatically added to Publons, you can then choose whether or not to claim your Publons credit. I understand this statement. Yes
